# H_2_O_2_‐Activated Serotonin Precursor Probe for Mapping Neuronal Redox Homeostasis Reveals 5‐HT Interactions with Neighboring Proteins Under Oxidative Stress

**DOI:** 10.1002/advs.202502360

**Published:** 2025-07-21

**Authors:** Yani Liu, Jiwen Yuan, Tuanjie Zhang, Xinyi Cai, Meng Xu, Xueao Wang, Rui Wang, Bing Zhang, Hai‐Liang Zhu, Yong Qian

**Affiliations:** ^1^ State Key Laboratory of Microbial Technology Jiangsu Collaborative Innovation Center of Biomedical Functional Materials, School of Chemistry and Materials Science Nanjing Normal University Wenyuan Road 1 Nanjing 210046 P. R. China; ^2^ State Key Laboratory of Pharmaceutical Biotechnology, School of Life Sciences Nanjing University Xianlin Road 163 Nanjing 210023 P. R. China; ^3^ College of Material and Chemical Engineering Tongren University Tongren Guizhou 554300 P. R. China; ^4^ Pingshan Translational Medicine Center Shenzhen Bay Laboratory Shenzhen 518118 P. R. China; ^5^ School of Pharmacy, Inflammation and Immune Mediated Diseases Laboratory of Anhui Province Anhui Medical University Hefei 230032 P. R. China; ^6^ Department of Radiology, Nanjing Drum Tower Hospital The Affiliated Drum Tower Hospital of Nanjing University Medical School Nanjing 210008 P. R. China

**Keywords:** epilepsy, oxidative stress, probe, serotonin

## Abstract

Serotonin (5‐HT) is a critical neurotransmitter that regulates various neurophysiological processes. However, the role of 5‐HT under oxidative stress remains largely unexplored. Here, the development of a novel intramolecular charge transfer (ICT)‐based fluorescent probe is reported, termed **HOP**, designed using a tandem sensing and labeling strategy. **HOP** is selectively activated by hydrogen peroxide (H_2_O_2_) in neuronal cells undergoing oxidative stress. Upon activation, **HOP** emits fluorescent signals and covalently cross‐links with nearby proteins, which not only anchors it to the local microenvironment to avoid diffusion of the fluorophore, but also simultaneously releases 5‐HT in situ. The locally released 5‐HT further interacts with nearby functional proteins such as myeloperoxidase (MPO) and sirtuin 1 (SIRT1), as confirmed through mass spectrometry analyses. Furthermore, **HOP** is employed in high‐throughput screening to identify the antioxidant, hesperidin, that is effective in modulating H_2_O_2_ levels and 5‐HT homeostasis. Additionally, the efficacy of **HOP** in detecting H_2_O_2_ distribution is validated in vivo and ex vivo using epileptic mouse models. This study presents a robust tool for precise imaging of H_2_O_2_ in living neuronal systems and for exploring 5‐HT‐associated protein modifications under oxidative stress, thus providing new avenues for investigating the role of serotonin in neurological disorders, such as epilepsy.

## Graphical abstract

**HOP**, an innovative ICT‐based fluorescent probe, visualizes H_2_O_2_ in stressed neurons while locally releasing serotonin (5‐HT) for real‐time protein interaction mapping. Its unique tandem sensing and labeling strategy reveals 5‐HT's role under oxidative stress, offering a powerful tool for neurophysiological research and therapeutic discovery.

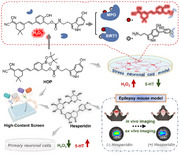

## 1 Introduction

Neurotransmitters are important signaling molecules that mediate neuronal communication and regulate diverse brain functions, thereby maintaining neurophysiological homeostasis.^[^ ^1^ ^]^ Among them, serotonin (5‐hydroxytryptamine, 5‐HT) is vital in regulating various neuropsychological processes in the central nervous system, including mood regulation, aggression, and memory.^[^ ^2^ ^]^ Serotonin exerts its effects mainly by activating 5‐HT receptors, which are widely distributed in the brain and primarily synthesized by neurons in the cerebral cortex and synapses, and extend to key regions such as the hippocampus.^[^ ^3^ ^]^ The different functions of 5‐HT are mediated through 12 different subtypes of G‐protein‐coupled receptors (GPCRs),^[^ ^4^ ^]^ which makes serotonin receptor agonists an important therapeutic option for the treatment of neurodegenerative disorders such as depression, schizophrenia, epilepsy, and Alzheimer's disease (AD).^[^ ^5^ ^]^ For example, serotonergic neurotransmission has been increasingly recognized for its significant role in epilepsy pathophysiology, as evidenced by clinical findings that indicate alterations in serotonin levels in patients with epilepsy. Increasing 5‐HT levels through pharmacological or neuromodulatory techniques can reduce seizure frequency and severity, which highlights the potential of targeting the serotonergic system as a new approach to epilepsy treatment. Beyond its receptor‐mediated actions, it is noteworthy that emerging evidence suggests that 5‐HT can also act outside of receptor activation,^[^ ^6^ ^]^ directly altering chromatin through post‐translational modifications targeting histones.^[^ ^7^ ^]^ These studies emphasize the critical role of the neurotransmitter 5‐HT in maintaining physiological homeostasis in the brain, and that antagonizing these functions or disrupting homeostasis can lead to deleterious effects.

Oxidative stress, a pathological hallmark of many neurological disorders, including epilepsy, arises from an imbalance between reactive oxygen species (ROS) production and the brain's antioxidant defenses, leading to lipid peroxidation, DNA oxidative damage, and neuroinflammation within neuronal cells.^[^ ^8^ ^]^ Serotonin has demonstrated antioxidative properties by suppressing excessive ROS production via activation of 5‐HT1A receptors, contributing to its neuroprotective effects.^[^ ^9^ ^]^ Moreover, serotonin plays a central role in modulating neuroimmune and neuroinflammatory responses, which are critical in the pathogenesis of epilepsy.^[^ ^10^ ^]^ Notably, 5‐HT has been shown to regulate the activity of microglia and astrocytes, two key cell types involved in neuroinflammation.^[^ ^11^ ^]^ Additionally, serotonin influences the activation, proliferation, and differentiation of T cells under inflammatory conditions.^[^ ^8^, ^12^ ^]^ The regulatory effects of serotonin on cytokine production vary by cell type, which further highlights the complexity of serotonin involvement in neuroinflammation. Pharmacological agents such as selective serotonin reuptake inhibitors (SSRIs) (e.g., fluoxetine) have anti‐inflammatory effects, further linking serotonin signaling to regulating chronic neuroinflammation.^[^ ^13^ ^]^ Despite these insights, there remains a limited understanding of how homeostatic changes of 5‐HT respond to oxidative stress in neuronal cells, particularly in the context of excessive ROS accumulation. The complex interplay between ROS, 5‐HT, and intracellular functional proteins under the pathological context of epilepsy remains poorly understood, which thus requires more in‐depth investigation to elucidate the underlying mechanisms. However, the lack of targeted research tools has significantly hindered progress in this area thereby expanding research methodologies and tools is crucial for advancing this field and unlocking new therapeutic avenues.

**Scheme 1:**
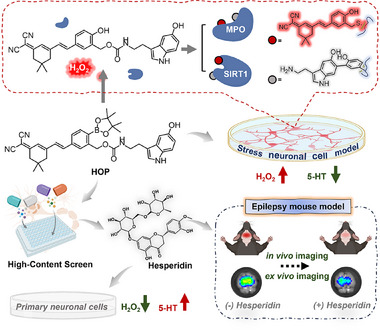
Design and chemical structure of **HOP**, a serotonin precursor probe for H_2_O_2_‐activated 5‐HT interaction with neighboring proteins under oxidative stress.

In this study, we report the development of an innovative chemical tool for probing neuronal redox homeostasis and elucidating the role of serotonin under oxidative stress, an intramolecular charge transfer (ICT)‐based fluorescent serotonin precursor, termed **HOP**. This new probe is selectively activated by hydrogen peroxide (H_2_O_2_), a key ROS biomarker prevalent in oxidative stress microenvironments within neuronal cells. The boronic acid moiety in **HOP** enables specific recognition of H_2_O_2_, triggering a fluorescence‐enhanced response that allows precise detection and tracking of H_2_O_2_ distribution in stressed neuronal cells. Upon activation, **HOP** releases a red fluorescent signal and covalently binds to cysteine residues on the surface of nearby proteins, thereby anchoring the fluorophore to the local microenvironment. Simultaneously, **HOP** facilitates the in situ release of serotonin, enabling localized interactions with neighboring proteins (**Scheme** **1**). This dual function not only restricts the diffusion of the fluorophore itself and improves the accuracy of intracellular H_2_O_2_ mapping, but it also facilitates the in situ investigations of 5‐HT interactions with neighboring proteins under such stressed conditions. The effectiveness of **HOP** in probing and imaging endogenous H_2_O_2_ fluctuations in different living neuronal cells, including primary microglia‐neuron co‐culture systems, was validated using flow cytometry and advanced microimaging techniques. With mass spectrometry, we further demonstrate that 5‐HT released from **HOP** under oxidative stress forms covalent modifications with tyrosine residues on the surface of nearby proteins, revealing novel insights into 5‐HT‐mediated protein interactions under oxidative stress. Moreover, we established a high‐throughput screening platform using **HOP** to identify therapeutic agents, leading to the discovery of hesperidin, a natural antioxidant that modulates H_2_O_2_ levels and 5‐HT homeostasis. Taking advantage of the excellent blood‐brain barrier permeability of **HOP**, we successfully applied this serotonin fluorescent precursor probe to in vivo and ex vivo brain imaging of H_2_O_2_ in epileptic mouse models. This work provides a novel platform for in situ monitoring H_2_O_2_ signaling in living primary neuronal cells and mouse models of epilepsy, as well as a new strategy for exploring aberrant protein modifications associated with 5‐HT under oxidative stress with controlled release of 5‐HT, which is expected to provide a powerful chemical tool for advancing imaging studies in models of other neurodegenerative diseases, such as AD.

## 2 Results and Discussion

### 2.1 Design and Synthesis of HOP

**Scheme 2:**
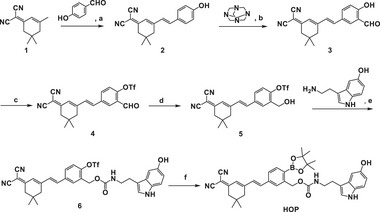
Synthesis of **HOP**. Reagents and conditions are the following: a) Piperidine, EtOH, 0 °C, 8 h; b) TFA, 72.4 °C, 2 h; c) DCM, Et_3_N, Tf_2_O, room temperature (rt), 30 min; d) MeOH, NaBH_4_, rt, 30 min; e) THF, CDI, 0 °C, 1.5 h; f) KOAc, Pd(dppf)Cl_2_, BinAP, DIO, 90 °C, 5 h.

The synthetic route to **HOP** is illustrated in **Scheme** **2**. Detailed experimental procedures and characterization data are available in the Supporting Information. Briefly, the reduction of intermediate compound 4 using sodium borohydride yielded alcohol‐functionalized compound 5, which was subsequently conjugated to the neurotransmitter 5‐hydroxytryptophan via a hydroxylamine condensation reaction, forming compound 6. In the final step, a palladium‐catalyzed cross‐coupling reaction introduced a boronic acid group via trifluoromethanesulfonyl substitution to yield the neurotransmitter precursor probe, **HOP**. Importantly, we speculated that the bis(cyanoisonitrile) ketoboronic acid alkyl derivatives synthesized in this study would have excellent fluorescence properties accompanied by significant photophysical properties.^[^ ^14^ ^]^ We hypothesized that under conditions of oxidative stress in neuronal cells, an abnormal elevation of ROS, particularly H_2_O_2_, would trigger activation of the **HOP** in the local microenvironment where intracellular stress is concentrated. Thanks to the free intracellular distribution and diffusion of the small‐molecule probe, this activation not only indicates the spatial and temporal distribution of intracellular oxidative stress through the release of fluorescence‐responsive signals, but also generates highly reactive electrophilic intermediates by converting boronic acid groups to the corresponding phenolic derivatives through activation. These intermediates would covalently bind to nucleophilic amino acid residues on nearby proteins. Such binding anchors the generated fluorophore to protein macromolecules, thereby restricting its diffusion and enhancing localization precision. Concurrently, the probe releases the active neurotransmitter 5‐HT into this local microenvironment (Figure S1, Supporting Information). This dual function of the **HOP** is particularly important, as it enables precise imaging of altered H_2_O_2_ homeostasis within the local microenvironment. Concurrently, the simultaneous release of 5‐HT in this local microenvironment offers valuable insights into whether there are specific interactions of 5‐HT with nearby proteins under oxidative stress conditions. These interactions may be critical in regulating physiological responses to oxidative stress, thus providing a new approach to understanding neurotransmitter dynamics and neurochemistry‐related pathophysiology in oxidative stress settings. Therefore, we envision that this innovative probe will be a powerful tool for us to study the spatial distribution of H_2_O_2_ signaling molecules and the local effects of 5‐HT under stress conditions, thereby contributing to a deeper understanding of oxidative stress mechanisms and neurotransmitter signaling in biological systems.

### 2.2 Spectroscopic Property Studies of HOP

**Figure 1:**
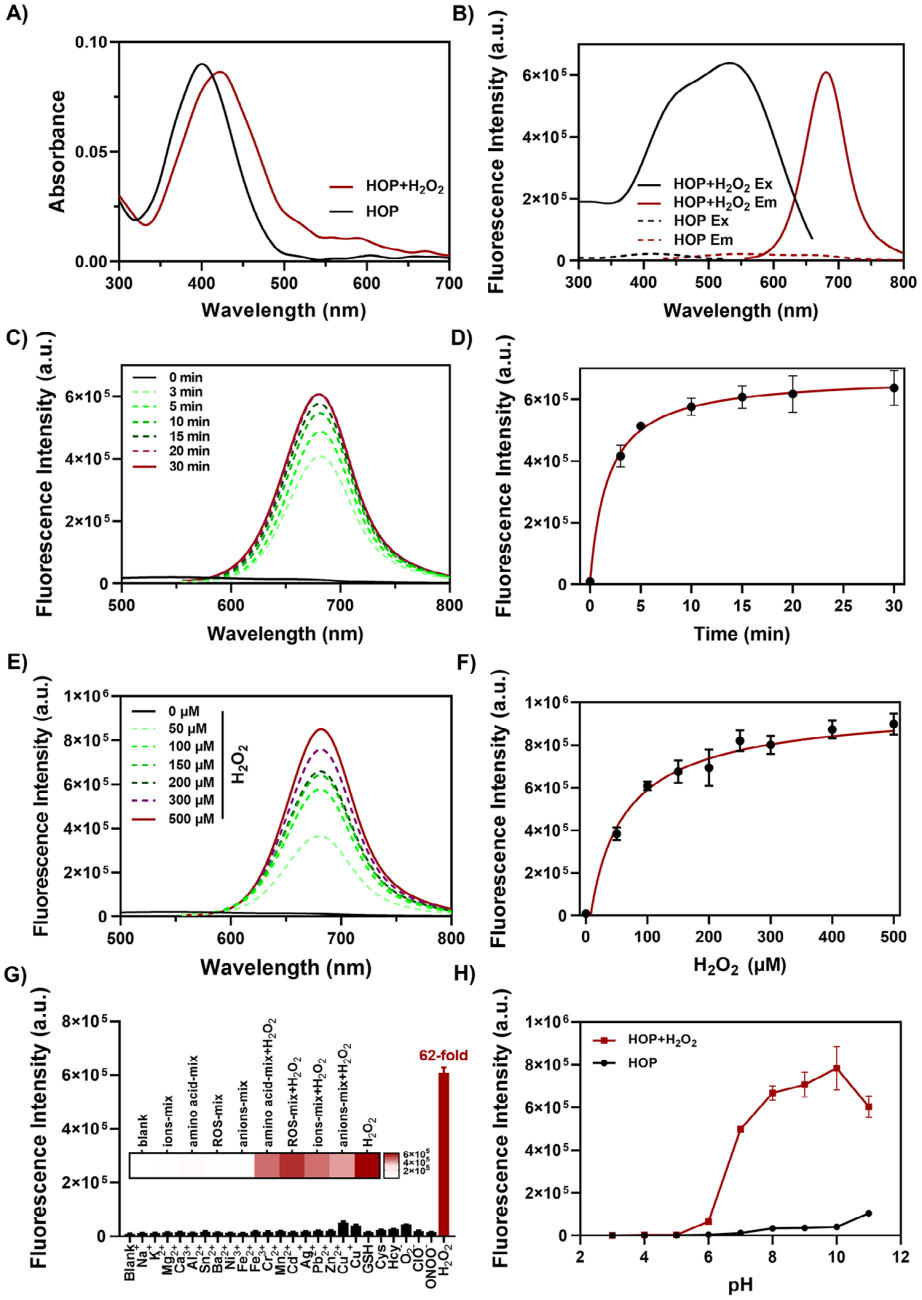
A) UV absorption spectra of **HOP** measured in PBS buffer (10 mM, pH 7.4, 1% DMSO with 1 mM CTAB). B) Fluorescence excitation and emission spectra of **HOP** (5 µM) before and after the addition of H_2_O_2_ (100 µM) in PBS buffer (10 mM, pH 7.4, 1% DMSO with 1 mM CTAB). C) Time‐dependent fluorescence spectra of **HOP** (5 µM) in the presence of H_2_O_2_ (100 µM) in PBS buffer (10 mM, pH 7.4, 1% DMSO, containing 1 mM CTAB) at 37 °C. Excitation: 561 nm, slit width: 5/5 nm. D) The kinetic profile of fluorescence intensity changes at 680 nm for **HOP** after H_2_O_2_ addition. E) Fluorescence emission spectra of **HOP** (5 µM) incubated with increasing concentrations of H_2_O_2_ (0‐500 µM) for 20 min. F) Concentration‐dependent fluorescence intensity change of **HOP** at 680 nm in response to H_2_O_2_. G) Fluorescence intensity of **HOP** (5 µM) at 680 nm following the addition of 100 µM of different species. Inset: The fluorescence intensity of **HOP** (5 µM) at 680 nm in the presence or absence of H_2_O_2_ after addition of various mixtures: 1) none, 2) ions‐mix (Na^+^, K^+^_,_ Mg^2+^, Ca^2+^, Al^3+^, Sn^2+^, Ba^2+^, Ni^2+^, Fe^3+^, Fe^2+^, Cr^3+^, Mn^2+^, Cd^2+^, Ag^+^, Pb^2+^, Zn^2+^, Cu^2+^, and Cu^+^), 3) amino acid‐mix (GSH, Cys, and Hcy), 4) ROS‐mix (^1^O_2_, ClO^−^, and ONOO^−^), 5) anions‐mix (NO_3_^−^, NO_2_^−^, SO_4_^2−^, F^−^, Cl^−^, Br^−^, HCO_3_^−^, HS^−^, S^2−^, AcO^−^, and SCN^−^), 6) amino acid‐mix and H_2_O_2_, 7) ROS‐mix and H_2_O_2_, 8) ions‐mix and H_2_O_2_, 9) anions‐mix and H_2_O_2_, 10) H_2_O_2_ alone. H) Fluorescence intensity of **HOP** (5 µM) at 680 nm in response to H_2_O_2_ (100 µM) under different pH conditions (3.0–11.0). All data represent the mean of three independent experiments.

With this new probe in hand, we first evaluated the reactivity of **HOP** with H_2_O_2_ in a methanol system using High‐Performance Liquid Chromatography in vitro. Upon addition of H_2_O_2_ to the **HOP** solution, a new peak emerged, whereas the original **HOP** peak diminished, suggesting that the probe undergoes conversion to a fluorescent product in the presence of H_2_O_2_ (Figure S2, Supporting Information). To explore whether our designed probe exhibits a fluorescence turn‐on response upon exposure to H_2_O_2_, we measured the fluorescence spectra of **HOP** before and after its reaction with H_2_O_2_ in Phosphate‐buffered saline (PBS) buffer. The probe itself displayed a distinct absorption peak at ≈410 nm (**Figure** **1**A). In the absence of H_2_O_2_, the emission peak of **HOP** was observed near 580 nm. However, after treatment with H_2_O_2_, a significant increase in fluorescence intensity at 680 nm was observed, accompanied by a red shift of 100 nm (Figure 1B). The fluorescence intensity increased by ≈60‐fold within 10 min, which would reach a plateau after 30 min (Figure 1C,D). Subsequently, we further evaluated the sensitivity of **HOP** toward H_2_O_2_. As the concentration of H_2_O_2_ increased, the fluorescence signal was gradually enhanced within the range of 0–200 µM (Figure 1E,F). In addition, the fluorescence spectra of **HOP** after incubation in solvents of different viscosities were also measured, and it was found that **HOP** showed a decrease in fluorescence intensity and a blue shift in the emission spectra at gradually increased viscosities (Figure S3, Supporting Information). Notably, at low H_2_O_2_ concentrations, the response demonstrated a clear linear relationship, with a LOD determined to be 0.41 µM (Figure S4, Supporting Information). The fluorescence quantum yield of **HOP** itself was calculated to be 0.21%, which increased to 1.2% upon reaction with H_2_O_2_. We determined the lipid solubility of **HOP** by measuring the UV absorption of **HOP** in a buffered solution of water and 1‐Octanol, and found that **HOP** had a logP value of 1.4382, suggesting favorable cell membrane permeability (Figure S5, Supporting Information). Additionally, we tested whether **HOP** had selective response properties, and we observed that **HOP** was more selective for H_2_O_2_ than other reactive oxygen species, metal cations, anions, and amino acids (Figure 1G). To assess the probe's suitability under physiological conditions, we evaluated its fluorescence intensity across different pH levels. The probe exhibited a significant fluorescence response in the pH range of 6.0 to 11.0 (Figure 1H). It may be due to the stronger nucleophilicity of the boronic acid group on the **HOP** under alkaline conditions and the enhanced electron‐donating ability of the phenolic hydroxyl group formed upon activation by H_2_O_2_.^[^ ^15^ ^]^ Together, these results suggest that **HOP** has strong potential for sensing and monitoring H_2_O_2_ levels in biological systems, offering both sensitivity and selectivity under physiological conditions.

### 2.3 HOP as a Dual‐Activity Probe for H_2_O_2_ Sensing and Protein Labeling Under Oxidative Stress

**Figure 2:**
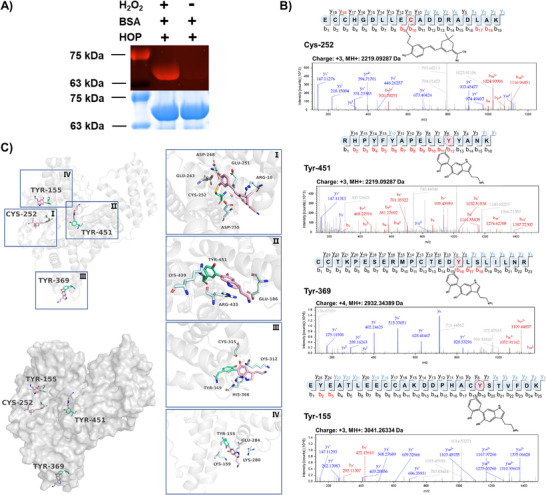
H_2_O_2_‐dependent labeling of **HOP** to BSA and identification of its binding sites by protein profiling. A) BSA (1 µg µL^−1^, 10 µL) was incubated with 50 µM **HOP** in the presence of 1 mM H_2_O_2_ for 20 min, then separated by SDS‐PAGE and analyzed by In‐gel fluorescence and Coomassie blue staining. B) Peptides modified by **HOP** were identified using mass spectrometry. Four key sequences (ECCHGDLLECADDRADLAK, RHPYFYAPELLYYANK, CCTYPESERMPCTEDYLSLILNR, and EYEATLEECCAKDDPHACYSTVFDK) suggest that Cys‐252, Tyr‐451, Tyr‐369, and Tyr‐155 are the primary binding sites on BSA. C) Computational simulation of modified binding sites on BSA demonstrating the overall site docking of activated **HOP** covalently binding to BSA. The simulated docking of the four covalent binding sites of BSA (Cys‐252, Tyr‐451, Tyr‐369, and Tyr‐155) was shown in I, II, III, and IV, respectively.

To determine whether **HOP** anchors to protein surfaces by cross‐linking with neighboring proteins upon its activation in the microenvironment in the presence of H_2_O_2_, we investigated the protein‐labeling behavior in the presence and absence of H_2_O_2_ using bovine serum albumin (BSA) as a model protein substrate in vitro. After the reaction system of **HOP** with proteins was placed in a microenvironment with different concentrations of H_2_O_2_ and HClO, the protein samples were separated by sodium dodecyl sulfate‐polyacrylamide gel electrophoresis (SDS‐PAGE) and analyzed by in‐gel fluorescence imaging (**Figure** **2**A; Figure S6, Supporting Information). As expected, a strong fluorescent band corresponding to BSA was observed only in the presence of H_2_O_2_, confirming that **HOP** specifically labels proteins in an H_2_O_2_‐dependent manner. This observation highlights the selectivity of the probe to the oxidative stress microenvironments and its ability to label nearby proteins under such conditions. To further elucidate the detailed mechanism of its protein labeling, we performed an identification study of the covalent modification sites by enzymatically digesting **HOP**‐treated BSA samples and analyzing them using mass spectrometry (Figure 2B). The results of the secondary mass spectrometry analysis confirmed that the fluorophore released from **HOP** covalently cross‐linked with cysteine residues on BSA in the presence of H_2_O_2_, while the released 5‐HT cross‐linked with tyrosine residues. We noted weak fragmentation ion signals between some of the peptides, which may be due to technical limitations of the DDA‐based mass spectrometry method, however, given the specificity of 5‐HT modification under oxidative stress (5‐HT specifically labels Tyr residue), and the fact that the analyzed peptide sequences (Figures 2B and 5E; Figure S26, Supporting Information) contained only a single Tyr residue (including Tyr155 and Tyr286, etc.), and there are no alternative modification sites in the sequences, thus we can confidently assign these residues as the 5‐HT modification sites. These findings suggest that **HOP** will be activated in the presence of H_2_O_2_ and that the released fluorophore and 5‐HT will covalently cross‐link with neighboring proteins in such an oxidative stress microenvironment. Furthermore, to visualize the spatial variation of the interaction between **HOP** and BSA, we performed molecular docking simulations, and the docking results indicated the covalent binding pattern of the probe upon H_2_O_2_ activation (Figure 2C). This computational analysis supports previous experimental results demonstrating that **HOP**, as an H_2_O_2_‐responsive fluorescent precursor, covalently anchors to neighboring proteins upon H_2_O_2_ activation, which facilitates the avoidance of free diffusion of fluorophores and thus will provide more precise spatio‐temporal information in live cell imaging studies. To verify that **HOP** releases 5‐HT in the cellular oxidative stress microenvironment, we performed HPLC assays of 5‐HT content in cells after incubating cells with **HOP** for different times and at different concentrations in the presence or absence of H_2_O_2_ (Figure S7, Supporting Information). The results of the analysis revealed that **HOP** released 5‐HT in cells in the presence of H_2_O_2_ only, and the content of 5‐HT increased gradually with the increase of the concentration, but the content of 5‐HT decreased after the incubation time reached 3 h, which may be due to the metabolism of the excess 5‐HT by the cells. Together, these results suggest that **HOP** acts as a dual‐function probe, both sensing H_2_O_2_ and labeling neighboring protein substrates under oxidative stress. This property helps to precisely track the spatiotemporal distribution of H_2_O_2_ signaling molecules in living cells. Moreover, **HOP** activation triggers the release of 5‐HT, facilitating the study of the interaction of 5‐HT with neighboring proteins under stress conditions, thus potentially providing insight into oxidative stress‐related processes such as neurotransmitter dysregulation.

### 2.4 HOP can Detect Changes in Endogenous and Exogenous H_2_O_2_ at the Cellular Level

**Figure 3:**
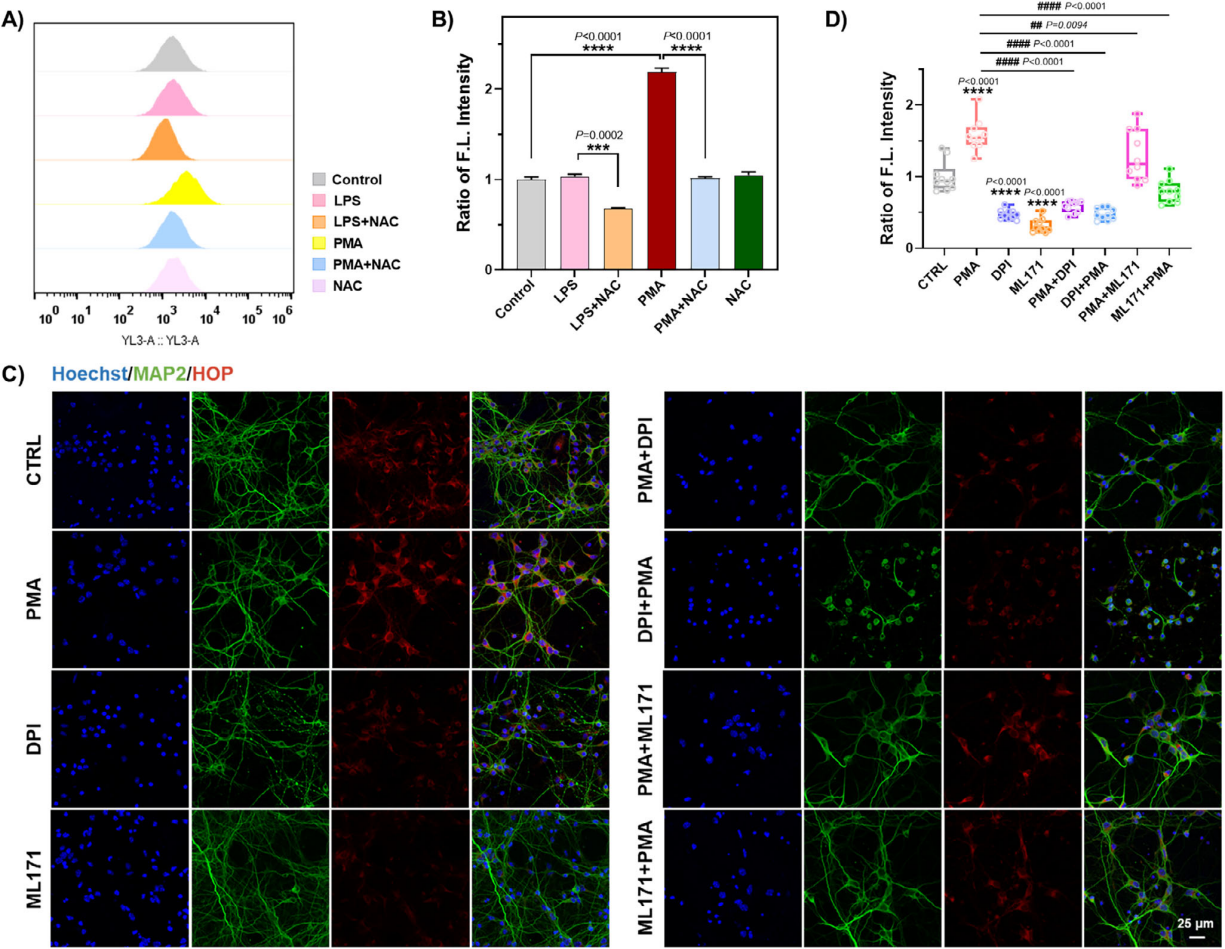
A) FACS analysis of living cell labeling of GL261 cells with **HOP** (5 µM). Living cells treated and untreated with LPS (1 µg mL^−1^, 12 h), PMA (1 µg mL^−1^, 1 h), and NAC (2 mM, 30 min), were incubated with **HOP** (5 µM) for 20 min at 37 °C and then analyzed by flow cytometry using YL3 channel (excitation: 561 nm, emission: 655–735 nm). B) Quantification of relative fluorescence intensities of the cells shown in A. C) Confocal imaging analysis of living primary neural cells labeled with **HOP** (5 µM). Living primary neural cells were treated with PMA (1 µg mL^−1^, 1 h), DPI (5 µM, 30 min), and ML171 (10 µM, 30 min), followed by incubation with **HOP** for 20 min at 37 °C. Fluorescence signals were collected from the emission channel at 650–750 nm with excitation at 561 nm. The excitation wavelength of Hoechst was 405 nm, and the emission collection range was 420–480 nm. The excitation wavelength of MAP2 was 488 nm, and the emission collection range was 500–550 nm. Scale bar: 25 µm. D) Quantification of relative fluorescence intensities of the cells shown in (C), with untreated cells set as 1. Statistical analyses were performed using one‐way ANOVA with multiple comparisons. *Means compared with the CTRL group. *p*  <  0.05 was considered significant (^*^ *p*  <  0.05, ^**^ *p*  <  0.01, ^***^ *p *<  0.001, ^****^ *p*  <  0.0001). Error bars represent ± SEM (*n* = 10).

Prior to the imaging studies, we evaluated the cytotoxicity profile of **HOP** itself on the living cells. GL261 cells were incubated with different concentrations of **HOP** (1‐10 µM), and no significant cytotoxic effects were observed (Figure S8, Supporting Information). Cells incubated with **HOP** maintained good photostability under continuous excitation for a long period of 300 s (Figure S9, Supporting Information). Therefore, **HOP** is suitable for imaging detection of intracellular H_2_O_2_. Next, we sought to investigate the responsiveness of **HOP** to both endogenous and exogenous H_2_O_2_ in a variety of living cell types. To this end, we first tested the ability of **HOP** to detect exogenous H_2_O_2_, flow cytometry and confocal imaging confirmed that **HOP** fluorescence intensity correlated with H_2_O_2_ concentration (Figures S10 and S11, Supporting Information). These findings suggest that **HOP** can effectively image exogenous H_2_O_2_ in live cells. Since the cellular environment is enriched with thiols, they may react with **HOP** as well as H_2_O_2_. We performed fluorescence imaging analysis of cells after treatment with different thiol small molecules and found that the fluorescence intensity of cells incubated with glutathione (GSH), cysteine (Cys), and hydrogen sulfide (H_2_S) was significantly lower than that of cells incubated with H_2_O_2_, showing a specific response to H_2_O_2_ in the physiological environment (Figure S12, Supporting Information). Subsequently, we extended the use of **HOP** to visualize endogenous H_2_O_2_ in various cell models under different stimulation conditions. Initial experiments focused on detecting H_2_O_2_ produced by GL261 cells under oxidative stress induced by lipopolysaccharide (LPS) and phorbol 12‐myristate 13‐acetate (PMA). Cells were treated with LPS (1 µg mL^−1^) for 12 h and PMA (1 µg mL^−1^) for 1 h to stimulate ROS generation, followed by **HOP** staining for 20 min. Analysis by flow cytometry and confocal imaging showed a significant increase in fluorescence in PMA‐stimulated cells (**Figure** **3**A,B; Figure S13, Supporting Information). The addition of N‐acetylcysteine (NAC, an H_2_O_2_ scavenger) inhibited this fluorescence enhancement, demonstrating the sensitivity of **HOP** for H_2_O_2_. Further experiments were performed using SH‐SY5Y cells to detect endogenous H_2_O_2__._ Given that NADPH oxidase (NOX) plays a key role in H_2_O_2_ production, we used the NOX inhibitors diphenyleneiodonium chloride (DPI) and 2‐acetylphenothiazine (ML171), as well as the antioxidant Ebselen, to inhibit the increase in endogenous H_2_O_2_ induced by LPS and PMA stimulation. Flow cytometry and confocal imaging confirmed that the fluorescence of cells was enhanced compared with control cells. This enhancement was markedly attenuated after pretreatment with DPI, ML171, or Ebselen, whereas the fluorescence of LPS‐ and PMA‐treated cells was effectively blocked by DPI and Ebselen (Figures S14 and S15, Supporting Information). Co‐localization experiments revealed that the fluorescent signals were primarily localized to the endoplasmic reticulum and mitochondria (Figure S16, Supporting Information). Finally, we evaluated the application of **HOP** in primary neuronal cells under PMA‐induced oxidative stress. Imaging of neurons after 1 h of PMA stimulation showed a corresponding increase in endogenous H_2_O_2_, reflected by significant fluorescence enhancement compared to controls. In contrast, treatment with DPI and ML171 significantly reduced both baseline fluorescence and PMA‐induced fluorescence enhancement (Figure 3C,D). These findings are consistent with those obtained in other cell models. Collectively, all these results confirm that **HOP**, a dual‐activity‐based probe, can effectively detect changes in both endogenous and exogenous H_2_O_2_ in living cells under stimulated conditions using microscopy and flow cytometry.

### 2.5 HOP Facilitates the Imaging of Endogenous H_2_O_2_ Production in Primary Neuronal Cells Under Glutamate and Kainic Acid

**Figure 4:**
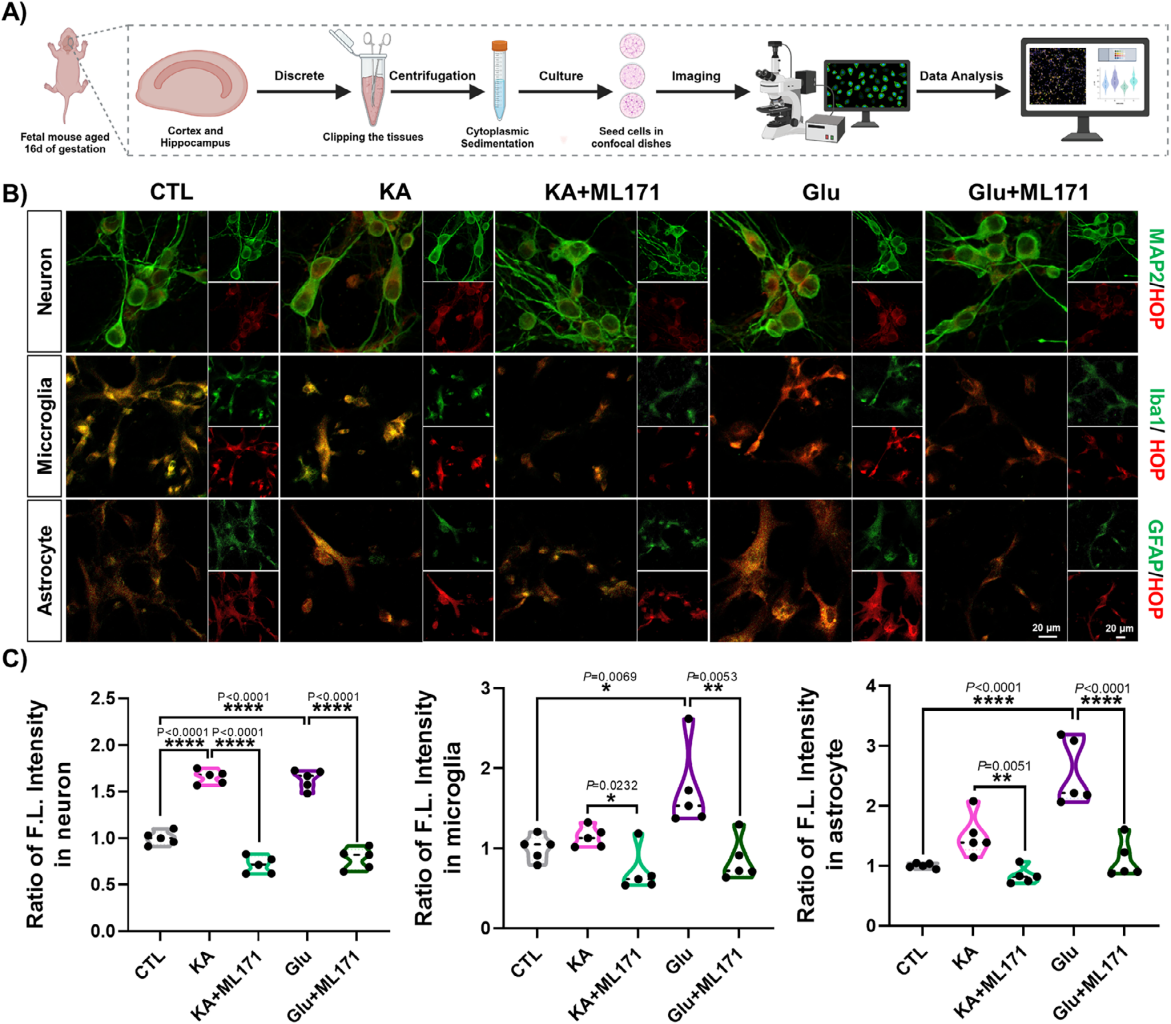
A) Schematic workflow for live‐cell labeling and imaging in primary cultures of mouse‐derived neurons, microglia, and astrocytes. B) Confocal imaging analysis of live‐cell labeling of primary neurons, microglia, and astrocytes using **HOP**. Living primary neuronal cells were treated with KA (500 µM, 1 h), Glu (2 mM, 1 h), and ML171 (10 µM, 30 min), followed by incubation with **HOP** (5 µM) at 37 °C for 20 min. Fluorescence signals of **HOP** were collected from the emission channel at 650–750 nm, with excitation at 561 nm. MAP2 (neuronal marker), Iba1 (microglial marker), and GFAP (astrocytic marker) were excited at 488 nm, with emissions collected in the 500–550 nm range. Scale bar: 20 µm. C) Quantification of the relative ratios of fluorescence intensities of the labeled cells shown in panel (B). Untreated cells were used as controls and set to 1. Statistical analyses were performed using one‐way ANOVA with multiple comparisons. Statistical significance was determined at *p* < 0.05 (^*^ *p* < 0.05, ^**^ *p* < 0.01, ^***^ *p* < 0.001, ^****^ *p* < 0.0001). Data are presented as mean ± SEM (n = 5).

Since **HOP** can effectively monitor intracellularly produced endogenous H_2_O_2_, we extended its application to study oxidative stress signaling in primary neuronal cells. Oxidative stress, particularly the accumulation of ROS like myeloperoxidase (MPO)‐associated H_2_O_2_, is thought to be closely associated with neurological disorders, such as epilepsy.^[^ ^16^ ^]^ To explore whether **HOP** can visualize the dynamics of endogenous H_2_O_2_ within the MPO protein microenvironment of primary neuronal cells under stimulated stress, we subsequently employed **HOP** to analyze H_2_O_2_ production in living primary neuronal cells. Previous reports have demonstrated that high levels of glutamate (Glu) can induce oxidative stress.^[^ ^17^ ^]^ To test this, we preloaded primary neurons, microglia, and astrocytes with glutamate and kainic acid (KA, a glutamate analog and widely used epilepsy‐inducing reagent)^[^ ^18^ ^]^ for 1 h, followed by co‐incubation with **HOP** for 20 min. Cells treated with glutamate and KA exhibited significantly brighter fluorescent signals compared to controls, indicating that **HOP** is highly sensitive to elevated oxidative stress. Similarly, the NADPH oxidase 1 inhibitor ML171 (**Figure** **4**A–C) and the MPO inhibitor 4‐ABAH (Figure S17, Supporting Information) prevented the enhancement of fluorescence. These findings provide direct evidence that primary neurons and glial cells produce excess endogenous H_2_O_2_ in response to stimulation with glutamate and its analog KA, and that our neurotransmitter precursor‐based probe, **HOP**, specifically recognizes and detects endogenous H_2_O_2_ produced in these primary neuronal cells.

### 2.6 HOP Enables the Direct Visualization of Transcellular H_2_O_2_ Exchange in a Microglia‐Neuron Co‐Culture Model

**Figure 5:**
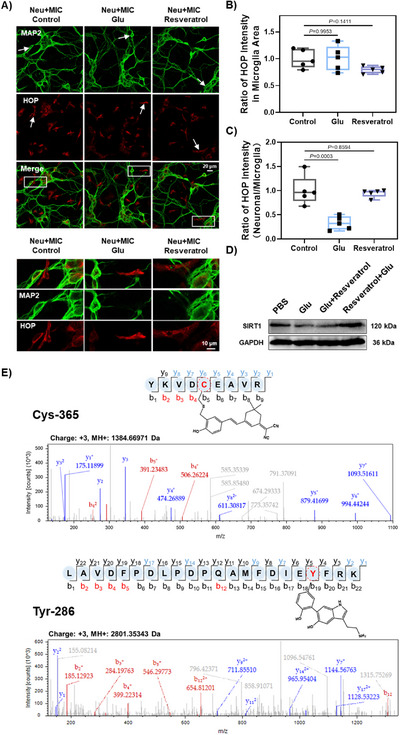
A) Confocal imaging of primary neurons co‐cultured with primary microglia. Cells were treated with Glu (2 mM, 1 h) and resveratrol (1 µM, 1 h), followed by incubation with **HOP** at 37 °C for 20 min. The fluorescence signals of **HOP** were collected within the emission channel at 650–750 nm, with excitation at 561 nm. MAP2, a neuronal marker, was excited at 488 nm, with emission collected between 500–550 nm. Scale bar: 20 µm. The scale bar in the amplified view of the white boxed area is 10 µm. B) Quantification of the fluorescence intensity of **HOP** in primary microglia in A. C) The ratio of **HOP** fluorescence intensity between primary neurons and microglia in A. The fluorescence intensity of the control group was normalized to 1. Statistical analyses were performed with one‐way ANOVA with multiple comparisons. Significance levels, ^*^ *p *< 0.05, ^**^ *p *< 0.01. Data are represented as mean ±SEM (*n* = 5). D) Western blot analysis of SIRT1 expression in HT22 cells co‐treated with Glu and resveratrol. E) Identification of peptides modified by mass spectrometry, YKVDCEAVR, and LAVDFPDLPDPQAMFDIEYFRK as the two major modifications of **HOP**, suggesting that Cys‐365 and Tyr‐286 are the binding sites for SIRT1.

Recent studies have indicated that the deacetylase SIRT1 plays a significant role in oxidative stress injury.^[^ ^19^ ^]^ We hypothesized a potential link between H_2_O_2_ signaling and SIRT1 activity. Previous work demonstrated that resveratrol, a known SIRT1 activator, exerts anti‐inflammatory effects by reducing the excessive accumulation of ROS.^[^ ^20^ ^]^ Therefore, we considered applying resveratrol as a potential modulator of H_2_O_2_ for chemical modulation or intervention in subsequent studies on transcellular signaling of H_2_O_2_. Using flow cytometry, we first assessed cells sequentially or simultaneously stimulated by Glu and resveratrol (Figure S18, Supporting Information). The results revealed a significant increase in fluorescence in Glu‐stimulated cells, indicative of elevated intracellular H_2_O_2_ levels, whereas resveratrol treatment attenuated this response. These findings suggest that resveratrol can alleviate abnormal intracellular H_2_O_2_ accumulation by chemically modulating neuronal cells. Building on the above findings demonstrating that **HOP** can monitor H_2_O_2_ production by various intracellular pathways, we subsequently sought to apply this molecular tool to investigate the feasibility of visualizing transcellular H_2_O_2_ signaling in a microglia‐neuron co‐culture system. Microglia, as specialized resident macrophages of the central nervous system (CNS), can be activated by Glu to produce H_2_O_2_ through the superoxide dismutase pathway.^[^ ^21^ ^]^ Thus, the co‐culture system offers an effective platform to study ROS transfer between different cell types under selective microglial activation. Meanwhile, we introduced resveratrol as an SIRT1 activator to inhibit abnormally elevated intracellular H_2_O_2_ levels. In this setup, microglia and neurons were co‐cultured, treated with Glu for 1 h, and then incubated with **HOP** for 20 min (**Figure** **5**A). A significant reduction in the fluorescence signal ratio of neurons/microglia in the Glu‐stimulated co‐cultures was observed, suggesting that Glu‐induced stress disrupted redox homeostasis in neuronal cells (Figure 5B,C). The observed increase in microglial H_2_O_2_ levels in response to neuronal stress confirmed that **HOP** effectively captures and monitors intracellular H_2_O_2_ flux, enabling the visualization of transcellular stress signaling between neurons and microglia in a complex co‐culture model. Conversely, we also observed a stabilization of the fluorescence ratio in the resveratrol‐treated experimental group, further suggesting that resveratrol eliminates excess H_2_O_2_. To further verify that the elimination of H_2_O_2_ by resveratrol occurs through SIRT1 activation, we performed Western blot analysis using a SIRT1‐specific antibody to assess its expression in response to Glu stimulation and the antagonistic effect of resveratrol. Results showed that Glu stimulation significantly reduced SIRT1 expression, whereas co‐treatment with resveratrol restored its levels (Figure 5D; Figure S19, Supporting Information). These findings suggest the role of resveratrol in SIRT1‐mediated antioxidant processes and demonstrate the utility of **HOP** as a versatile probe for tracking dynamic H_2_O_2_ flux in neuronal cells. Taken together, **HOP** provides a robust platform for directly observing H_2_O_2_ signaling in complex neuronal systems, offering valuable insights into the interplay between oxidative stress, SIRT1 activity, and the neuroprotective effects of resveratrol.

### 2.7 High‐Throughput Screening of Natural Products Using HOP

Given the robust performance of **HOP** in live‐cell imaging and its sensitivity to oxidative stress induced by Glu and KA, we subsequently explored its potential as a tool for high‐throughput screening of antioxidants that modulate H_2_O_2_ homeostasis. To this end, we developed a fluorescence‐based high‐throughput screening platform integrating high‐content screening (HCS) and high‐content analysis (HCA). Live HT22 neuronal cells were pretreated with KA for 1 h to induce oxidative stress, followed by incubation with 20 µM of various natural products with potential antioxidant properties for another 1 h. After incubation with 5 µM **HOP**, endogenous H_2_O_2_ was imaged and quantified using HCA (Figure S20, Supporting Information). This approach ensured rapid detection and efficient quantification of endogenous H_2_O_2_. The results demonstrated that most of the selected natural products induced a significant decrease in **HOP** fluorescence signal, suggesting a downregulation of H_2_O_2_ production. Furthermore, we performed flow cytometric analyses that further confirmed the reduction in H_2_O_2_ levels (Figure S21, Supporting Information). Hesperidin, a classical flavonoid with multiple biological activities,^[^ ^22^ ^]^ effectively inhibited **HOP** fluorescence compared with untreated negative controls and KA‐pretreated positive controls. This identifies hesperidin as a promising candidate for modulating H_2_O_2_ homeostasis in live cells. Notably, this study demonstrates the application of **HOP** as a direct screening tool for identifying lead compounds capable of regulating endogenous H_2_O_2_ levels in neuronal cells. Following the HCA screening, we extended our investigation to primary neuronal cells subjected to high concentrations of oxidative stress inducers, including Glu and KA. Parallel experiments were conducted with cells treated with hesperidin and the SIRT1 activator resveratrol (Figures S22 and S23, Supporting Information). Imaging of the Glu‐ and KA‐stimulated cells revealed enhanced fluorescence, indicating elevated H_2_O_2_ levels compared to controls. In contrast, cells treated with hesperidin or resveratrol exhibited a significant reduction in fluorescence, suggesting the efficacy of these compounds in mitigating oxidative stress. These observations reveal that hesperidin exerts potent antioxidant effects in neuronal cells. Collectively, these results highlight the utility of **HOP** as a high‐throughput screening tool for identifying natural products that modulate oxidative stress in live neuronal cells. The ability of hesperidin to reduce H_2_O_2_ levels presents new opportunities for developing therapeutic strategies targeting oxidative stress‐related neuronal damage.

### 2.8 HOP Precursors Release 5‐HT Under Oxidative Stress and Modify Neighboring Functional Proteins

Our previous studies have demonstrated that **HOP** selectively detects elevated levels of intracellular H_2_O_2_ under oxidative stress conditions induced by various stimuli. Notably, a screening effort identified hesperidin, a natural product with strong antioxidant properties, which effectively reduced elevated H_2_O_2_ levels in KA‐stimulated neuronal cells. Next, we sought to gain insight into whether 5‐HT under oxidative stress can label functional proteins in the microenvironment in which it resides and whether the regulatory compound hesperidin facilitates the restoration of 5‐HT homeostasis. MPO and SIRT1 have been recognized as critical regulatory proteins that influence 5‐HT receptor activity, especially in neurodegenerative diseases.^[^ ^23^ ^]^ Based on these insights, we subsequently investigated the interactions between neurotransmitter precursor **HOP**, MPO, and SIRT1 under conditions that mimic abnormal levels of H_2_O_2_, aiming to elucidate potential covalent modifications of these selected functional proteins by **HOP**. Using an H_2_O_2_‐dependent assay, we incubated **HOP** with MPO or SIRT1 proteins (Figures S24 and S25, Supporting Information) and then analyzed the protein labeling after the reaction by SDS‐PAGE. Fluorescent bands corresponding to MPO and SIRT1 were observed in the presence of H_2_O_2_, indicating selective protein modification. Mass spectrometry further confirmed that the fluorophore portion of **HOP** covalently bound to cysteine residues in both MPO and SIRT1, whereas the 5‐HT portion formed covalent bonds with tyrosine residues in these functional proteins (Figure 5E; Figure S26, Supporting Information). Similarly, a detailed molecular docking study provided structural validation of these covalent interactions, revealing the precise binding configurations of **HOP** with MPO and SIRT1, respectively, following activation by H_2_O_2_ (Figures S27 and S28, Supporting Information).

Moreover, immunofluorescence co‐localization imaging of **HOP** with MPO and SIRT1 also showed that **HOP** was relatively well‐localized with proteins (Figures S29 and S30, Supporting Information). These findings suggest that under oxidative stress, **HOP** is covalently anchored to protein macromolecules in the local microenvironment, facilitating the release of the neurotransmitter 5‐HT through interactions with neighboring proteins. This mechanism mirrors the dynamic alterations in redox homeostasis observed in stressed intracellular environments, where aberrant interactions between 5‐HT precursors and functional proteins under such oxidative stress microenvironments may disrupt normal neurotransmitter function, potentially contributing to the onset and progression of neurodegenerative diseases. More importantly, this process inevitably leads to a significant depletion of intracellular 5‐HT, indicating a critical disruption of its homeostasis under oxidative conditions. To further investigate the homeostatic changes of 5‐HT during oxidative stress and the potential role of modulators in regulating its homeostasis, we measured 5‐HT levels in KA‐stimulated cells and compared them with cells treated with hesperidin. As expected, 5‐HT levels were significantly reduced in KA‐treated cells, whereas hesperidin treatment partially restored 5‐HT levels (Figures S31 and S32, Supporting Information). Taken together, all these observations suggest the dual role of **HOP** as both a sensor and a responsive neurotransmitter precursor, providing an important tool for probing 5‐HT‐related processes during oxidative stress and valuable insights into oxidative stress mechanisms.

### 2.9 In Vivo Imaging of H_2_O_2_ Flux in the Brain of Epileptic Mice

**Figure 6:**
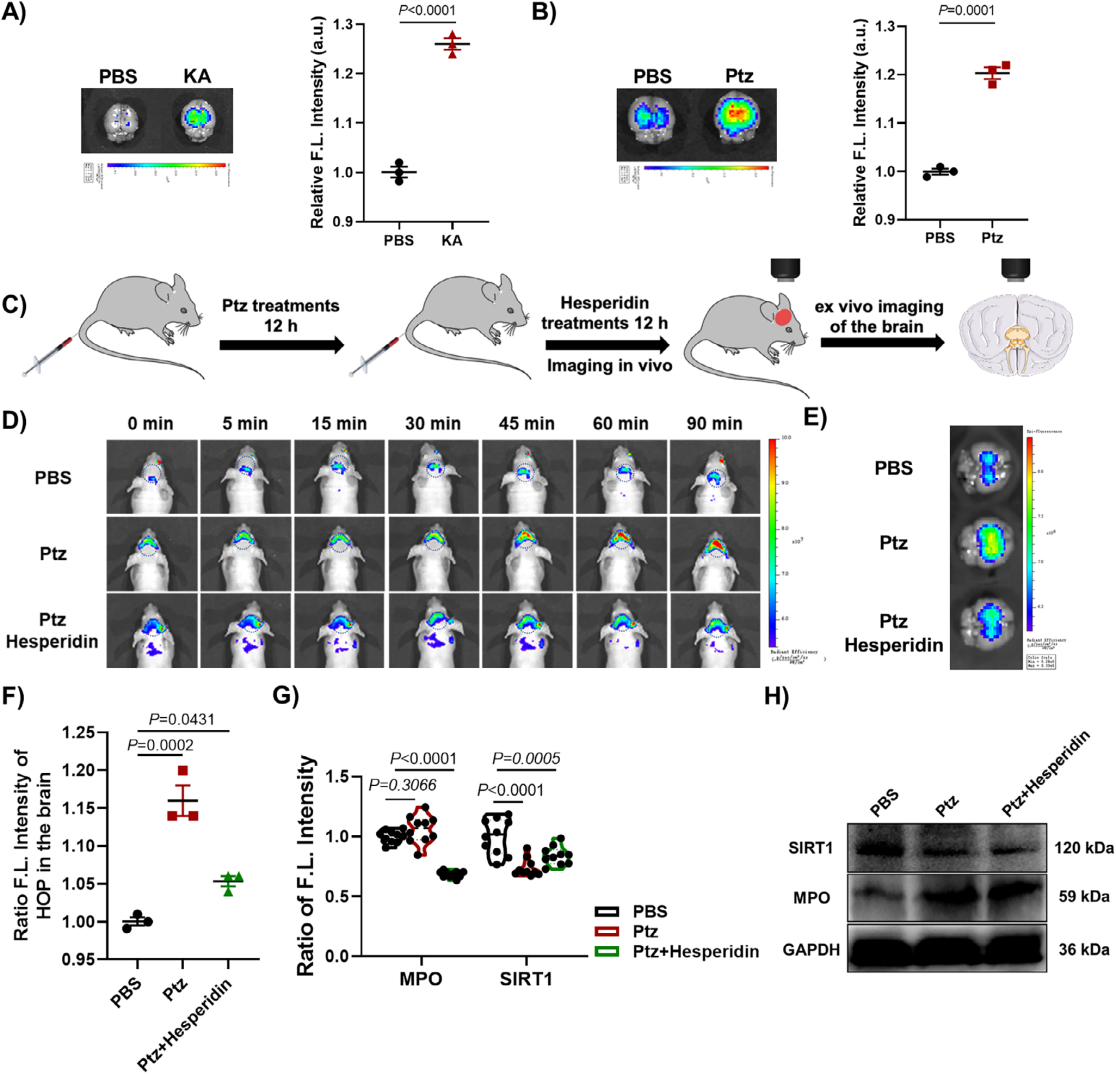
In vivo and ex vivo fluorescence imaging of H_2_O_2_ levels in the brains of epileptic mice and after hesperidin treatment. A) Ex vivo fluorescence images of H_2_O_2_ signals in the brains of KA‐induced epileptic mice after 60 min postinjection of **HOP** using the IVIS Spectrum imaging system. Fluorescence signals in the PBS‐treated control group were normalized to 1. B) Ex vivo fluorescence images of H_2_O_2_ signals in the brains of Ptz‐induced epileptic mice after 60 min postinjection of **HOP** using the IVIS Spectrum imaging system. Fluorescence signals in the PBS‐treated control group were normalized to 1. C) Schematic diagram of in vivo and *ex vivo* brain imaging in Ptz‐induced epileptic mice after treatment with Hesperidin. D) In vivo fluorescence images were acquired at 5, 15, 30, 45, 60, and 90 min after intravenous injection of **HOP** into mice pre‐stimulated with Ptz (60 mg kg^−1^) and treated with hesperidin (20 mg kg^−1^) for 12 h. Excitation: 560 nm, Emission: 680 nm. E) Ex vivo fluorescence images showing H_2_O_2_ fluorescence signals in the brains of control and treated mice after 90 min postinjection of **HOP** using the IVIS Spectrum imaging system. F) Quantification of the relative ratios of fluorescence intensities of the brains shown in E. Fluorescence signals in the PBS group were normalized to 1. Statistical analyses were performed with one‐way ANOVA with multiple comparisons. ^*^ *p *< 0.05, ^**^ *p *< 0.01. Error bars represent ±SEM (*n *= 3). G) Quantification of the relative ratios of fluorescence intensities of the brain tissues shown in Figure S36 (Supporting Information). Fluorescence signals in the PBS group were normalized to 1. Statistical analyses were performed with one‐way ANOVA with multiple comparisons. ^*^ *p *< 0.05, ^**^ *p *< 0.01, ^***^ *p *< 0.001, ^****^ *p *< 0.0001. Error bars represent ±SEM (*n* = 10). H) The expression levels of MPO and SIRT1 in hippocampal tissues of treated and control mice were detected by Western blotting.

Finally, to evaluate the potential of **HOP** as a tool for in vivo imaging of endogenous H_2_O_2_ fluxes in a neurological disease model, we established a KA‐induced epilepsy model in BALB/c nude mice via intraperitoneal injection of a low dose of KA (6 mg kg^−1^), a widely used mode for studying seizures.^[^ ^24^ ^]^ Following tail vein injection of **HOP** (3 mg kg^−1^), in vivo imaging revealed significantly elevated fluorescence intensity in the brains of epileptic mice at different time points (0, 10, 20, 30, 45, and 60 min) compared to the PBS control group. This increase in fluorescence indicates that **HOP** effectively crossed the blood‐brain barrier and tracked the upregulation of H_2_O_2_ in the brain (Figure S33, Supporting Information). Furthermore, ex vivo fluorescence imaging of the brain tissue of these mice validated these in vivo observations, confirming the accuracy of the **HOP**‐based detection of oxidative stress (**Figure** **6**A). To further validate the reliability of the **HOP** application, we employed the pentylenetetrazole (Ptz)‐induced acute epilepsy mouse model, another classical model of seizures.

Like the KA model, **HOP** was able to detect elevated H_2_O_2_ levels in the epileptic mouse brain under oxidative stress conditions induced by Ptz (Figure 6B; Figure S34, Supporting Information). Consistent with previous findings, the fluorescence signal in the brain was significantly enhanced in the epilepsy model group, which was higher than in the PBS group. Together, these in vivo and ex vivo studies demonstrate that **HOP** exhibits excellent imaging performance and holds promise as a tool for real‐time detection of dynamic changes in endogenous H_2_O_2_ in the brain of living epilepsy mouse models.

To further explore whether hesperidin can exert antioxidant effects in an epileptic mouse model, we utilized **HOP** imaging to observe its effects in classical Ptz‐induced epileptic mice, in which a high dose of Ptz (60 mg kg^−1^) was administered intraperitoneally to wild‐type mice to induce acute epilepsy. Following intravenous administration of hesperidin (3 mg kg^−1^), in vivo imaging showed a noticeable attenuation of brain fluorescence signals in the hesperidin‐treated epileptic mice compared to those treated with Ptz alone (Figure 6C,D; Figure S35, Supporting Information). Ex vivo brain imaging further validated these findings (Figure 6E,F). These results suggest that hesperidin effectively modulates the overproduction of H_2_O_2_ in the brain of epileptic mice, offering valuable insights into the modulation of oxidative stress in the brain during pharmacological treatment of epilepsy. Moreover, to elucidate the underlying molecular mechanisms, we investigated the expression of SIRT1 and MPO proteins, key markers in oxidative stress regulation, in the brains of Ptz‐treated mice and hesperidin‐cotreated states (Figure S36, Supporting Information). Immunofluorescence staining of brain sections revealed that Ptz‐induced epilepsy was associated with increased MPO expression and decreased SIRT1 expression. In contrast, hesperidin treatment resulted in a significant upregulation of SIRT1 and a concomitant reduction in MPO levels (Figure 6G). These findings suggest that reduced SIRT1 activity is associated with elevated MPO expression, which in turn exacerbates H_2_O_2_‐mediated oxidative stress. Fortunately, hesperidin appears to counteract this process by activating SIRT1, inhibiting MPO, and scavenging excess H_2_O_2_. In addition, protein immunoblotting of brain tissue from the cerebral cortex and hippocampus confirmed these results, exhibiting that hesperidin treatment upregulated SIRT1 expression while downregulating MPO (Figure 6H; Figures S37 and S38, Supporting Information). Collectively, these findings suggest that intracerebral stress in epileptic mice leads to up‐regulation of MPO and concomitant reduction of SIRT1 activity, and that hesperidin may be a potent activator of SIRT1 to antagonize this process. Our newly designed neurotransmitter precursor **HOP** can visualize these changes and regulation of H_2_O_2_ homeostasis in the brain during this process, providing a robust chemical tool for studying oxidative stress regulation in neurological disease models. This study highlights the utility of **HOP** for real‐time monitoring of oxidative stress in vivo and underscores the potential of hesperidin as a therapeutic agent for modulating oxidative stress pathways in epilepsy.

## 3 Conclusion

In this work, we present a novel serotonin precursor fluorescent probe, **HOP**, designed for the accurate detection of intracellular oxidative stress and the controlled release of 5‐HT in neuronal cells. The probe leverages an intramolecular charge transfer mechanism and responds to H_2_O_2_, making it highly suitable for real‐time monitoring of oxidative stress signaling. **HOP** exhibits several advantageous properties, including non‐toxicity, good photostability, and excellent biocompatibility. As a small molecule, it diffuses freely throughout the cellular environment, allowing for comprehensive stress detection. However, once **HOP** reacts with H_2_O_2_, the diffusion of the postresponse fluorophore is limited due to its anchoring to nearby protein macromolecules in the local microenvironment, thus providing spatial precision in detecting intracellular H_2_O_2_ distribution. Importantly, **HOP** enables the detection of endogenous H_2_O_2_ fluxes in various cell types, including the study of transcellular signaling between microglia and neurons in co‐culture models. Under oxidative stress, **HOP** is activated by H_2_O_2_, leading to the generation of a fluorophore that covalently binds to neighboring proteins, while simultaneously releasing 5‐HT into the localized microenvironment. Interestingly, our observations also reveal that 5‐HT interacts with nearby proteins in such oxidative stress microenvironments, contributing to specific protein labeling. Notably, we identified a new mechanism by which 5‐HT interacts with key functional proteins such as MPO and SIRT1 under oxidative stress, deepening our understanding of the role of 5‐HT in neuroinflammation and neurological disorders. The dual functionality of **HOP**, both as a fluorescent probe and a modulator of 5‐HT release, provides a unique platform for studying oxidative stress in neurological diseases such as epilepsy. Moreover, we developed a simple screening method combining high‐throughput analysis, which led to the identification of hesperidin as a potent antioxidant modulator capable of attenuating oxidative stress in the brain and restoring 5‐HT homeostasis to some extent. In vivo and ex vivo imaging studies in an epilepsy mouse model demonstrated that **HOP** successfully tracked endogenous H_2_O_2_ fluxes in the brain. Further immunofluorescence and immunoblotting analyses of brain tissue confirmed that hesperidin modulated the expression of MPO and SIRT1 through its antioxidant effects. The successful application of **HOP** in a live animal model highlights its potential as a valuable tool for mapping oxidative stress in epileptic brains. These observations not only deepen our understanding of intracellular H_2_O_2_ distribution during oxidative stress in different neuronal cells but also provide new insights into the role of 5‐HT in protein post‐modification under oxidative stress conditions. We believe that by expanding the research toolkit for studying the role of serotonin in oxidative stress, this work provides unique tools and opens new avenues and opportunities for studies targeting neurological disorders.

## 4 Experimental Section

### Materials and Instrumentation

Reagents were purchased from Sigma Aldrich or Alfa Aesar. All chemicals and solvents were of analytical reagent grade and were used as received without further purification. Complete DMEM medium, fetal bovine serum (FBS), phosphate‐buffered solution (PBS), trypsin‐EDTA, and penicillin‐streptomycin were obtained from Nanjing SenBeiJia Biological Technology Co., Ltd (Nanjing, China). The synthesis of all compounds used in this study is outlined in Scheme 2. Compounds used for modulating endogenous H_2_O_2_ levels are listed in Table S1 (Supporting Information). All reactions requiring anhydrous conditions were performed under an N_2_ atmosphere using AR‐grade dry solvent (water content ≤ 30 ppm), and reactions were carried out in dry glass bottles. Reaction progress was monitored by thin‐layer chromatography (TLC) on Merck silica gel aluminum plates (GF‐254), with visual detection achieved using UV light or staining. ^1^H and ^13^C nuclear magnetic resonance (NMR) spectra were recorded on a Bruker DRX400 (400 MHz), or Bruker DRX600 (600 MHz). Data were given in the following order: chemical shifts were reported in parts per million (ppm) referenced concerning appropriate internal standards or residual solvent peaks (CDCl_3_: δ = 7.26 ppm for ^1^H, δ = 77.16 ppm for ^13^C; DMSO‐*d*_6_: δ = 2.50 ppm for ^1^H, δ = 39.52 ppm for ^13^C); multiplicities were indicated as follows: br s (broad singlet), s (singlet), d (doublet), dd (double doublet), t (triplet), q (quartet), m (multiplet); coupling constant values were given in Hertz (Hz). High‐resolution mass spectra (HR‐MS) were performed using an LTQ Orbitrap mass spectrometer coupled with an Accela HPLC‐System (flow injection: 50% water (containing 0.1% formic acid) and 50% acetonitrile (containing 0.1% formic acid), flow rate: 250 µL min^−1^). Ultraviolet absorption spectra were recorded on a UV spectrometer UV2550. Fluorescence measurements were carried out on a Hitachi F‐7000 fluorescence spectrophotometer with a slit width set to 5 nm. The emission spectrum was scanned from 600 to 800 nm at a scan rate of 1200 nm min^−1^, with the photomultiplier voltage set at 650 V.

### General Procedure for Spectral Analysis

Unless otherwise required, all the fluorescence spectra were performed according to the following procedure. In a 1.5‐mL centrifuge tube, 2 µL of the stock solution (1 mM) of **HOP**, 2 µL of the stock solution (100 mM) of CTAB, and an appropriate volume of PBS (pH 7.4) were added, followed by the addition of an appropriate amount of H_2_O_2_. The mixed solution was adjusted to 200 µL with H_2_O. After incubation at 37 °C for 20 min, the reaction solution was transferred to a quartz cell, and the fluorescence was measured (λ_ex/em_ = 535/680 nm; slit widths = 5 nm). For absorbance measurements, 200 µL of the reaction solution was prepared and used. Meanwhile, the probe itself, without H_2_O_2,_ was prepared as a blank control and measured under the same conditions for comparison.

### The Limit of Detection (LOD) of HOP

The emission spectrum of free **HOP** in PBS buffer (10 mM, pH = 7.40, 1 mM CTAB, containing 1% DMSO) was collected 20 times to confirm the background noise σ. The linear regression curve was then fitted according to the data in the range of H_2_O_2_ from 0 to 20 µM and obtained the slope of the curve, then the limit of detection was calculated using the following equation: The limit of detection (LOD) = 3σ/k, where σ is the standard deviation of blank measurements, and k is the slope of the linear equation. The LOD of HOP was then determined to be 0.406 µM.

### Determination of the Fluorescence Quantum Yields (Φ)

The relative fluorescence quantum yields of **HOP** were estimated by comparing the areas under fluorescence spectra of the test samples with that of the standard sample, where rhodamine B (10 µM, Φ = 0.69, λ_ex_ = 365 nm) was used as the standard sample and reference. The fluorescence quantum yields were calculated according to the following equation:

(1) $$\Phi_{x}=\left[A_{s}/A_{x}\right]\left[{n_{x}}^{2}/{n_{s}}^{2}\right]\left[F_{x}/F_{s}\right]\Phi_{s}$$

where *s* = standard; *x* = sample; *A* = absorbance at the excitation wavelength; *n* = refractive index; and *F *= area under the fluorescence spectra on an energy scale. The optical properties of these probes (10 µM) were examined in EtOH. Rhodamine B was used as a calibration standard under absorbance‐matched conditions at 365 nm.

### The Measurement of Lipophilicity (Log Po/w)

The lipophilicity was presented as a log Po/w value, which was determined by the flask‐shaking method. An aliquot of a stock solution of the probe **HOP** in aqueous NaCl (0.9% w/v and saturated with 1‐Octanol) was added to an equal volume of 1‐Octanol (saturated with 0.9% NaCl, w/v), and the mixture was shaken for 3 days to allow for partitioning at 298 K. After centrifugation of the samples at 134 000 rpm for 10 min, the probe content of the organic and aqueous phases was determined by fluorescence spectroscopy. Log Po/w was defined as the logarithmic ratio of probe concentrations in the organic and aqueous phases.

### In‐Gel Protein Fluorescence Analysis

**HOP** was incubated at 37 °C for 30 min in a buffer containing 10 mM sodium phosphate (pH 7.4), supplemented with H_2_O_2_ and selected proteins, including bovine serum albumin (BSA), myeloperoxidase (MPO), and sirtuin 1 (SIRT1). Protein samples were loaded onto 8% or 10% SDS‐PAGE protein gel converted (80 V, 30 min) and separated (120 V, 1–2 h). Following electrophoresis, the protein bands of interest were excised from the gel and cut into small pieces. Then, the gel pieces were destained with 50% acetonitrile (ACN)/50 mM ammonium bicarbonate (NH_4_HCO_3_). The gel pieces were incubated with 55 mM iodoacetamide in 25 mM NH_4_HCO_3_ to alkylate the digested protein fragments. Subsequently, the gel fragments were digested with 200 ng Trypsin (Promega Sequence Grade Modified) in 50 mM NH_4_HCO_3_ for 4 h. The resulting peptide recovery solution was desalted using home‐prepared C18 tips, and the treated peptide samples were analyzed by liquid chromatography‐tandem mass spectrometry (LC‐MS/MS).

### MS Analysis

**HOP** was incubated at 37 °C for 30 min in a buffer containing 10 mM sodium phosphate (pH 7.4) and supplemented with H_2_O and different proteins (BSA, MPO, and SIRT1). Following the reaction, samples were reconstituted in 8 µL of 0.1% formic acid and separated by an Easy‐nLC 1200 liquid chromatography system (Thermo Fisher Scientific) coupled to an Orbitrap Exploris 480 mass spectrometer through a nanoelectrospray ion source (Thermo Fisher Scientific). For peptide separation, 1 µg of peptides was injected into a reverse‐phase C18 column (75 µM i.d. × 15 cm, 3 µM particle size) and eluted at a flow rate of 250 nL min^−1^. Separation was achieved using a linear gradient of acetonitrile in a 0.1% formic acid over 90 min. Full‐scan MS spectra (m/z 350–1800) were acquired with a resolution of 60000 following ion accumulation to a predictive automatic gain control (AGC) target of 3×10^6^. The 12 most intense ions were sequentially selected for higher‐energy collisional dissociation (HCD) fragmentation with a normalized collision energy of 28. MS/MS spectra were acquired at a resolution of 15 000 with an AGC target of 1×10^5^. A dynamic exclusion time was set to 30 s.

### Peptide and Protein Identification

Raw mass spectrometry data were processed and analyzed using Proteome Discoverer 2.4 (PD2.4, Thermo Fisher Scientific) and searched against the UniProt/Swiss‐Prot database. The database search allowed up to two missed cleavage sites by trypsin. The peptide tolerance was set to 10 ppm, and MS/MS tolerance was set to 0.02 Da. Fixed modifications included carbamidomethylation of cysteine, while variable modifications included methionine oxidation, etc. The peptides with low confidence scores, as defined by PD2.4, were filtered out, and the remaining peptides were considered for peptide identification and possible binding site determinations. All MS/MS spectra for possibly identified peptides from initial database searching were manually inspected and validated using Proteome Discoverer (version 2.4) software.

### Cell Culture

SH‐SY5Y and GL261 cells were cultured in Dulbecco's Modified Eagle's medium (DMEM) supplemented with 10% fetal bovine serum (FBS) and 2% antibiotics. Cells were grown at 37 °C in a humidified incubator with 5% CO_2_/95% air.

### Cytotoxicity Assay

The cytotoxic effects of **HOP** on GL261 cells were evaluated using an MTT assay. GL261 cells were seeded into 96‐well plates at a density of 5000 cells per well and allowed to adhere. Cells were then treated with varying concentrations of **HOP** (0, 0.01, 0.1, 1, 10, and 100 µM) and incubated for 24 h under standard culture conditions (37 °C, 5% CO_2_). Subsequently, cells were treated with 10 µL of 5 mg mL^−1^ MTT and incubated for another 4 h (37 °C, 5% CO_2_). After incubation, the supernatant was carefully discarded, and 150 µL of DMSO was added to solubilize the formazan crystals. The plate data was read at 570 nm with a reference wavelength of 630 nm using an Infinite M200 Pro Multi‐Mode Microplate Reader (Tecan, Switzerland).

### Flow Cytometry Analysis

Before FACS analysis, GL261 cells were incubated with fresh medium containing **HOP** (5 µM) for 20 min and washed twice with PBS. After cell digestion and isolation, cells were centrifuged at 1000 rpm for 5 min, resuspended once in PBS, and centrifuged again at 1000 rpm for another 5 min. The resulting cells were resuspended in 500 µL of PBS and then placed on ice for measurement. Cellular fluorescence signals were collected using a BD FACSCalibur flow cytometer (FACS Calibur, Becton Dickinson (BD) Biosciences, San Jose, CA, USA). Three independent experiments were performed, and the resulting data were analyzed using FlowJo software.

### Fixed‐Cell Imaging

Cells in the logarithmic growth phase were inoculated into 12‐well plates containing glass slides and cultured overnight. Cells were pre‐treated with DPI (5 µM) or Ebselen (5 µM) in the presence of LPS (1 µg mL^−1^) or PMA (1 µg mL^−1^) diluted in 2% DMEM high‐glucose medium. Cells were then incubated in a humidified incubator at 37 °C with 5% CO_2_ for 30 min. Subsequently, the test compound **HOP** (5 µM) was added to the medium, and the cells were incubated for an additional 20 min at 37 °C. Following treatment, cells were washed twice with PBS and fixed with 4% paraformaldehyde. Fluorescence imaging was performed using a Zeiss LSM 880 inverted laser confocal microscope equipped with Airyscan. Imaging was conducted using an excitation wavelength of 561 nm and an emission acquisition range of 650–750 nm.

### Co‐Localization Imaging Studies of Living Cells

GL261 cells were incubated with 5 µM **HOP** for 20 min at 37 °C under 5% CO_2_. After incubation, cells were washed three times with 1× PBS and then co‐stained with ER‐Tracker Blue (1 µM, 30 min), Lyso‐Tracker Blue (60 nM, 60 min), and Mito‐Tracker Deep Red (100 nM, 30 min), respectively. Following staining, cells were rinsed twice with PBS and imaged in Hanks' balanced salt solution (1×, phenol red‐free, pH 7.2‐7.4). Fluorescence imaging was performed using a Zeiss LSM 880 inverted laser confocal microscope equipped with Airyscan (Nanjing University, Nanjing, China). Fluorescence channels were acquired as follows: the **HOP** signal was captured between 510–560 nm with excitation at 561 nm, the tracking blue channel was acquired between 410–600 nm with excitation at 405 nm, and the deep red channel was acquired between 650–700 nm with excitation at 633 nm. Images were analyzed using Image J software.

### Primary Neuron Culture

The hippocampus tissue of embryonic day 15.5 mouse embryos was dissected and chopped into ≈1 mm^3^ pieces in ice‐cold PBS. The hippocampus tissue was then digested in 10 mL of 0.1× trypsin (Gibco, 25200‐072) in PBS at 37 °C for 30 min. To reduce the viscosity of the cell‐tissue mixture, repeated pipetting was then performed. The digestion was neutralized by adding Neurobasal medium (Gibco, 21103‐049) supplemented with 2% B27 (Gibco, 17504‐044), 1% penicillin‐streptomycin (Sbjbio, BC‐CE‐007), and 1% GlutaMAX (Gibco, 35050–061). The suspension was filtered through a 40‐µm cell strainer (Corning, 431752) to separate single cells from tissue debris. Cells were centrifuged at 1500 × g for 10 min at 4 °C and gently resuspended in a Neurobasal medium supplemented with 2% B27, 1% penicillin‐streptomycin, and 1% GlutaMAX. After this, the cells were plated on 20‐mm‐diameter glass coverslips, which had been pre‐coated with 20 µg mL^−1^ poly‐d‐lysine (Sigma, P6407) in PBS. Coverslips were prepared by incubation in a 12‐well plate at 37 °C for 4 h before cell seeding. Cells were cultured in a humidified incubator at 37 °C with 5% CO_2_ for 10 days, with half of the medium replaced every 4 days.

### Primary Astrocyte Culture

Primary mouse astrocytes were isolated from the hippocampus of postnatal day 0 (P0) C57BL/6 mice, following a previously reported protocol (*Shao, W*. et al*., Nature 2013, 494, 90–94*). Mouse hippocampus tissue was dissected and chopped into ≈1 mm^3^ pieces in PBS on ice. The hippocampus tissue was then digested in 10 ml of 0.1× trypsin (Gibco, 252000–072) in PBS at 37 °C for 30 min. After digestion, repeated pipetting was performed to reduce the viscosity of the cell‐tissue suspension. DMEM/F12 medium (Gibco, C11330500BT) supplemented with 10% FBS (AusGeneX, FBS500‐S) and 1% penicillin‐streptomycin (HyClone, SV30010) was added to neutralize the trypsin. A 40‐µm cell strainer (431752, Corning) was used to separate cells from tissues. Cells were centrifuged at 1500×g for 10 min at 4 °C and gently resuspended in DMEM/F12 medium supplemented with 10% FBS and 1% penicillin‐streptomycin. After this, the cells were seeded into flasks and maintained in a humidified incubator at 37 °C with 5% CO_2_. After 24 h, the medium was replaced, and subsequent medium changes were performed every 4 days.

### Primary Microglia Culture

Microglia were isolated from the confluent astrocyte culture. Flasks containing astrocyte cultures were tapped and shaken vigorously to detach the microglia growing on top of the astrocyte layer. The floating microglial cells were collected, centrifuged, and resuspended in a glia‐conditioned medium. Microglia were either replated on poly‐D‐lysine (PDL)‐coated plates at a density of 2 × 10⁵ cells mL^−1^ or seeded on top of neuronal cultures at a density of 1 × 10⁵ cells mL^−1^.

### High Connotation Analysis (HCA)

Living HT22 cells (2×10^4^ cells/well) were seeded into the PerkinElmer CellCarrier‐96 plates and pretreated with Kainic acid (KA, 500 µM) for 1 h. Cells were then treated with various anti‐inflammatory agents (20 µM) or different antioxidants (20 µM) for 1 h, followed by incubation with **HOP** (5 µM) and the nuclear dye Hoechst (1 µg mL^−1^) for 20 min. After treatment, cells were washed three times with PBS to remove excess reagents. HCA was performed using a fluorescence imaging system. Imaging and quantitative analysis were performed with an excitation filter at 561 nm and an emission collection range of 650–720 nm.

### Preparation for the Standard Curve of the 5‐HT Control Solution

The appropriate amount of 5‐hydroxytryptamine (5‐HT) was weighed and dissolved in a solution of water/methanol/formic acid (98/2/0.2, v/v/v), according to its solubility and stability, to prepare a 100 µg mL^−1^ stock solution, and stored at −20 °C for spare use. To prepare the working standard solutions, 1 mL of the 5‐HT stock solution was mixed with a 50% methanol‐water solution and then diluted to a final volume of 10 mL. The mixture was well‐mixed to yield a 10 µg mL^−1^ standard control solution. Serial dilutions were then performed using the 50% methanol‐water solution to formulate the mixed control solution with concentrations of 5, 2, 1, 0.5, 0.2, 0.1, and 0.05 µg mL^−1^, respectively. Each control solution was reconstituted before use. A standard curve of 5‐HT was constructed by plotting the peak area from HPLC analysis against the concentration. The prepared homogenates of primary neuronal cells from different treatments were used as the test samples for quantification.

### Detection of 5‐HT Content in Primary Neuronal Cells

The well‐grown primary neuronal cells were incubated with KA for 12 h, followed by incubation with or without hesperidin for an additional 12 h. After treatment, the cells were removed from the incubator, washed twice with PBS, harvested by adding 1 mL of purified water, and scraped off with a cell scraper. The cell suspension was centrifuged at 1500 rpm for 8 min to collect the cell pellet. The supernatant was discarded, and the cell pellet was resuspended in 200 µL of purified water. Cell lysis was achieved using the freeze‐thaw method, and the sample was stored at −80 °C until further analysis. For the detection of 5‐HT, a 50 µL aliquot of the cell homogenate was mixed with 20 µL of trifluoroacetic acid (TFA) to precipitate proteins. The mixture was vortexed for 30 s and left at room temperature for 5 min. After incubation, the sample was centrifuged at 14000 rpm for 20 min at 4 °C. The supernatant was collected and centrifuged again for 10 min, and then the final supernatant was carefully collected and analyzed by HPLC.

### Animal Ethics

All mice used in this study were purchased from the Model Animal Research Centre of Nanjing Normal University (Nanjing, China). Mice were maintained under specific pathogen‐free conditions and used in accordance with the animal experimental guidelines established by the Institute of Animal Care and Use Committee. This study was approved by the Institutional Animal Care and Use Committee of Nanjing Normal University (IACUC‐2024270). Kainic acid (KA) and pentetrazol (Ptz)‐induced seizures in 5‐week‐old BALB/c nude mice were a widely used mouse model for epilepsy. The acute epilepsy model was induced by intraperitoneal injection (i.p.) of KA (6 mg kg^−1^) or Ptz (60 mg kg^−1^). In vivo and ex vivo fluorescence images of relative H_2_O_2_ levels in the mouse brain were further analyzed using an IVIS spectral imaging system (Nanjing University, China) at time points of 0, 10, 20, 30, 45, and 60 min after the injection of **HOP**. For the treatment group, mice were administered the antioxidant hesperidin (20 mg kg^−1^) for 12 h before intravenous injection of **HOP** (3 mg kg^−1^). In vivo and ex vivo fluorescence images of relative H_2_O_2_ levels in the mouse brain were further analyzed 0‐, 5‐, 15‐, 30‐, 45‐, 60‐, and 90‐min post‐**HOP** injection using the IVIS spectral imaging system (Nanjing University, China). The excitation filter wavelength was set at 560 nm, with an acquisition wavelength range of 650–720 nm.

### Preparation of Frozen Brain Tissue Sections

Brains were harvested and immediately frozen in liquid nitrogen following stereo imaging. For sectioning, the tissues were embedded in the optimal cutting temperature compound (OCT) after rewarming at −20 °C. Cryosectioning was performed using a Leica CM1950 cryostat at the optimal cutting temperature. Each section was ≈20 µm in thickness, dried, and stored at −80 °C in a refrigerator.

### Immunofluorescence

The frozen tissue sections were taken out and restored to room temperature before being fixed with freshly prepared 4% paraformaldehyde in 1× PBS for 10 min. The sections were then washed three times with PBS, each wash lasting 5 min. To permeabilize the tissue, 0.3% Triton X‐100 made in PBS was applied for 5–10 min, followed by three 5‐min washes with PBS. The tissue sections were blocked with the blocking buffer (3% BSA in 1× PBS) for 30–60 min. Primary antibodies (anti‐MPO, Proteintech, 22225‐1‐AP, 1:500; anti‐SIRT1, Proteintech, 60303‐1‐Ig, 1:100) were applied in a blocking buffer and incubated overnight at 4 °C. Afterward, the tissue sections were washed three times with 0.1% Tween 20 in PBS, each wash for 5 min. Alexa Fluor 488 Goat Anti‐Rabbit IgG (H+L) (Abclonal, AS073) and Alexa Fluor 488 Goat Anti‐Mouse IgG (H+L) (Beyotime, A0428) were applied in blocking buffer at a 1:500 dilution and incubated for 1 h at 37 °C. The tissue sections were washed again three times with 0.1% Tween 20 in PBS, followed by three washes with PBS, each lasting 5 min. Finally, a drop of anti‐fluorescence quenching sealant was applied before the sections were covered with cover glasses. Microscopic imaging was performed using a Leica TCS SP8 MP confocal fluorescent microscope. The immunofluorescence channel was excited at 488 nm, with the emission collected in the range of 500–550 nm.

### Western Blotting

Protein samples were separated by SDS‐PAGE on 8% or 10% gels. Initially, the samples were concentrated at 80 V for 30 min, followed by electrophoresis at 120 V for 1–2 h. After separation, proteins were transferred to a PVDF membrane using a Bio‐Rad Trans‐Blot wet blotting system for 60–90 min at 200 mA (buffer: 25 mM Tris, 192 mM Glycine, 20% MeOH, pH 8.3). The membrane was blocked with 5% nonfat dried milk in PBST (PBS with 0.1% Tween 20) for 1 h at room temperature. Following blocking, the membrane was washed once with PBST for 5 min and then incubated with specific primary antibodies (anti‐MPO, Proteintech, 22225‐1‐AP, 1:2000; anti‐SIRT1, Proteintech, 60303‐1‐Ig, 1:10000, and anti‐GAPDH, ABclonal, AC001, 1:20000) diluted in 5% milk in PBST. The incubation was performed for 1.5 h at room temperature or overnight at 4 °C. After primary antibody incubation, the membrane was washed three times for 10 min each with TBST and subsequently incubated with HRP‐conjugated Affinipure Goat Anti‐Rabbit IgG (H+L) (Proteintech, SA00001‐2, 1:5000) and HRP‐conjugated Affinipure Goat Anti‐Mouse IgG (H+L) (Proteintech, RGAM001, 1:5000) diluted in 5% milk in TBST for 1 h at room temperature. Western blot signals were detected using Tanon‐5200 Multi or Bio‐Rad ChemiDoc MP after incubating with ECL for 1 min.

### Statistical Analysis

Data analysis was performed using GraphPad Prism 8. Each data group was normalized based on the control group, and statistical tests performed included two‐tailed paired or unpaired Student's t‐tests, one‐way ANOVA, or two‐way ANOVA. All data are presented as mean ± S.E.M. Significance was set at *p < 0.05, **p < 0.01, ***p < 0.001 or ****p < 0.0001. Sample sizes were not pre‐determined using statistical methods, and all data distributions were assumed to be normal, but were not formally tested. For quantification, areas in cells and brain slices were randomly selected from regions of interest. Experimental steps were randomized to minimize the effects of confounding variables, including the way experimental mice were selected and the order of treatment. The researchers kept the sample information confidential. No data were excluded from analyses.

## Conflict of Interest

The authors declare no conflict of interest.

## Supporting information



Supporting Information

## Data Availability

The data that support the findings of this study are available from the corresponding author upon reasonable request.
