# Supplementary material for: H2O2‐Activated Serotonin Precursor Probe for Mapping Neuronal Redox Homeostasis Reveals 5‐HT Interactions with Neighboring Proteins Under Oxidative Stress
Source: Adv Sci (Weinh). 2025 Jul 21;12(39):e02360. doi: 10.1002/advs.202502360 (PMC12533203; doi:10.1002/advs.202502360)
Supplement: Supplementary file 1 — Supporting Information [file ADVS-12-e02360-s001.docx]

***Supporting Information***

**H₂O₂-activated serotonin precursor probe for mapping neuronal redox homeostasis reveals 5-HT interactions with neighboring proteins under oxidative stress**

Yani Liu^a,b,‡^, Jiwen Yuan^c,‡^, Tuanjie Zhang^d^, Xinyi Cai^b^, Meng Xu^a^, Xueao Wang^e^, Rui Wang^d^, Bing Zhang^f^, Hai-Liang Zhu^b^, Yong Qian^a,b,f,*^

[a] Y. Liu, M. Xu, Prof. Y. Qian
Jiangsu Collaborative Innovation Center of Biomedical Functional Materials, School of Chemistry and Materials Science
Nanjing Normal University
Wenyuan Road 1, Nanjing 210046, China
Email: yongqian@nju.edu.cn

[b] Y. Liu, X. Cai, Prof. H. Zhu, Prof. Y. Qian
State Key Laboratory of Pharmaceutical Biotechnology, School of Life Sciences
Nanjing University
Xianlin Road 163, Nanjing 210023, China

[c] J. Yuan
College of Material and Chemical Engineering
Tongren University
Tongren, Guizhou 554300, China

[d] T. Zhang, R. Wang
Pingshan Translational Medicine Center
Shenzhen Bay Laboratory
Shenzhen 518118, China

[e] X. Wang
School of Pharmacy, Inflammation and Immune Mediated Diseases Laboratory of Anhui Province
Anhui Medical University
Hefei 230032, P.R. China

[f] Prof. B. Zhang, Prof. Y. Qian
Department of Radiology, Nanjing Drum Tower Hospital
The Affiliated Drum Tower Hospital of Nanjing University Medical School
Nanjing 210008, China

[^‡^] These authors contributed equally.

**Contents**

1. ***Chemical Synthesis***
2. ***Supplementary Tables and Figures***
3. ***NMR Spectra & Mass Spectra***
4. ***Chemical*** ***Synthesis***

**Synthesis of** **Compound 2：**

A mixture of 4-hydroxy-benzaldehyde (5 g, 41 mmol) and 2-(3, 5, 5-trimethylcyclohex-2-enylidene) malononitrile (7.6 g, 41 mmol) was dissolved in a small volume of ethanol and heated under reflux in the presence of a catalytic amount of piperidine (3–4 drops) for 8h. Upon completion of the reaction, as confirmed by thin-layer chromatography (TLC), the solvent was removed under reduced pressure. The crude product was purified by recrystallization from ethanol to afford the desired compound **2** (3.6 g, 30.2% yield). ^1^H NMR (400 MHz, CDCl₃) δ 7.44 (d, *J* = 8.6 Hz, 2H), 7.01 (s, 1H), 6.88 (d, *J* = 8.5 Hz, 3H), 6.82 (s, 1H), 2.61 (s, 2H), 2.48 (s, 2H), 1.10 (s, 6H). ^13^C NMR (101 MHz, CDCl₃) δ 157.32, 154.44, 136.89, 129.42, 127.01, 122.75, 113.71, 112.95, 32.06, 28.05.

**Synthesis of Compound 3:**

To a solution of DCI-OH (3.6 g, 12.4 mmol) in trifluoroacetic acid (50 mL), hexamethylenetetramine (3.48 g, 12.4 mmol) was added. The reaction mixture was heated under reflux for 2 hours until the starting material was completely consumed, as confirmed by LC-MS analysis. After cooling to room temperature, the reaction mixture was poured into ice water (100 mL) and the resulting precipitate was collected by filtration. The crude product was purified by column chromatography using petroleum ether (PE) and ethyl acetate (EA) in a 1:1 ratio, yielding a red solid, identified as compound **3** (3.0 g, 75.8% yield). ^1^H NMR (400 MHz, CDCl₃) δ 11.19 (s, 1H), 9.96 (d, *J* = 0.4 Hz, 1H), 7.74 (dd, *J* = 6.3, 2.3 Hz, 2H), 7.08-7.02 (m, 2H), 6.94 (d, *J* = 16.2 Hz, 1H), 6.87 (s, 1H), 2.63 (s, 2H), 2.49 (s, 2H), 1.11 (s, 6H). ^13^C NMR (101 MHz, CDCl₃) δ 196.31, 169.13, 162.58, 153.39, 135.36, 134.79, 132.99, 128.39, 128.06, 123.59, 120.72, 118.75, 113.44, 112.70, 42.97, 39.22, 32.07, 28.05.

**Synthesis of Compound 4:**

A solution of (E)-2-(3-(3-formyl-4-hydroxystyryl)-5,5-dimethylcyclohex-2-en-1-ylidene)malononitrile (3 g, 9.4 mmol) in dichloromethane (DCM, 100 mL) was prepared and cooled to 0 °C in an ice bath. Triethylamine (1.31 mL, 9.4 mmol) was added, followed by trifluoromethanesulfonic anhydride (1.57 mL, 9.4 mmol) under stirring. The reaction mixture was allowed to warm to room temperature and stirred for an additional 30 minutes. To quench the reaction, a saturated solution of NaHCO₃ was added. The aqueous layer was extracted with DCM (3×50 mL), and the combined organic layers were dried over anhydrous MgSO₄, filtered, and concentrated under reduced pressure. The crude product was purified via column chromatography using a 1:1 mixture of PE and EA as the eluent, affording the desired compound **4** as an orange solid (3.2 g, 75.5% yield). ^1^H NMR (600 MHz, DMSO-*d_6_*) δ 10.09 (s, 1H), 8.44 (d, *J* = 2.3 Hz, 1H), 8.13 (dd, *J* = 8.7, 2.3 Hz, 1H), 7.67 (d, *J* = 8.6 Hz, 1H), 7.60 (d, *J* = 16.2 Hz, 1H), 7.41 (d, *J* = 16.2 Hz, 1H), 6.97 (s, 1H), 2.65 (s, 2H), 2.56 (s, 2H), 1.03 (s, 6H). ^13^C NMR (151 MHz, DMSO-*d_6_*) δ 189.25, 170.66, 155.15, 137.98, 135.29, 134.34, 133.18, 132.89, 128.98, 124.75, 123.98, 114.04, 113.27, 42.70, 38.58, 27.90.

**Synthesis of Compound 5:**

A solution of (E)-4-(2-(3-(dicyanomethylene)-5,5-dimethylcyclohex-1-en-1-yl)vinyl)-2-formylphenyl trifluoromethanesulfonate (3.2 g, 7.1 mmol) in MeOH (50 mL) was cooled to 0 °C. Sodium borohydride (NaBH₄, 269.6 mg, 7.1 mmol) was added portion-wise under stirring. The reaction mixture was allowed to warm to room temperature and stirred for 30 min. To quench the reaction, water was added, and the aqueous phase was extracted with DCM (3×50 mL). The combined organic layers were dried over anhydrous MgSO₄, filtered, and concentrated under reduced pressure. The resulting crude product was purified by column chromatography using a 2:1 mixture of PE and EA as the eluent, yielding compound **5** as a yellow solid (2.0 g, 62.3% yield). ^1^H NMR (600 MHz, DMSO-*d_6_*) δ 7.95 (d, *J* = 1.9 Hz, 1H), 7.76 (dd, *J* = 8.6, 2.1 Hz, 1H), 7.48 (d, *J* = 16.2 Hz, 1H), 7.43 (d, *J* = 8.6 Hz, 1H), 7.35 (d, *J* = 16.2 Hz, 1H), 6.96 (s, 1H), 4.61 (s, 2H), 2.63 (s, 2H), 2.56 (s, 2H), 1.03 (s, 6H). ^13^C NMR (151 MHz, DMSO-*d_6_*) δ 170.79, 155.67, 147.17, 135.85, 135.74, 131.84, 129.06, 128.73, 124.15, 122.09, 114.18, 77.74, 58.13, 42.75, 38.60, 32.14, 27.89.

**Synthesis of Compound 6:**

A solution of (E)-4-(2-(3-(dicyanomethylene)-5,5-dimethylcyclohex-1-en-1-yl)vinyl)-2-(hydroxymethyl)phenyl trifluoromethanesulfonate (2 g, 4.42 mmol) in tetrahydrofuran (THF, 35 mL) was cooled to 0 °C. Carbonyldiimidazole (CDI, 716 mg, 4.42 mmol) was added in one portion, and the reaction mixture was stirred at 0 °C for 30 min. A solution of 3-(2-aminoethyl)-1H-indol-5-ol (778 mg, 4.42 mmol) in THF (10 mL) was then added, and the reaction mixture was stirred for an additional 1 hour at room temperature. After completion, the reaction mixture was partitioned between ethyl acetate and water. The organic layer was washed sequentially with water and brine, dried over MgSO₄, and concentrated under reduced pressure. The resulting crude product was purified by silica gel column chromatography using a 1:1 mixture of PE and EA as the eluent, yielding compound **6** as an orange solid (1.0 g, 34.6% yield). ^1^H NMR (600 MHz, DMSO-*d_6_*) δ 10.48 (s, 1H), 8.60 (s, 1H), 7.96 (s, 1H), 7.85 (d, *J* = 10.7 Hz, 1H), 7.53 (d, *J* = 8.6 Hz, 1H), 7.49 (d, *J* = 16.2 Hz, 1H), 7.43 (t, *J* = 5.7 Hz, 1H), 7.33 (d, *J* = 16.2 Hz, 1H), 7.12 (d, *J* = 8.6 Hz, 1H), 7.03 (s, 1H), 6.94 (s, 1H), 6.82 (d, *J* = 2.0 Hz, 1H), 6.59 (dd, *J* = 8.6, 2.2 Hz, 1H), 5.11 (s, 2H), 3.27-3.22 (m, 2H), 2.77-2.72 (m, 2H), 2.64 (s, 2H), 2.55 (s, 2H), 1.02 (s, 6H). ^13^C NMR (151 MHz, DMSO-*d_6_*) δ 170.79, 155.67, 147.17, 137.06, 135.85, 135.74, 131.84, 129.06, 128.73, 124.15, 122.09, 119.60, 114.18, 113.35, 77.74, 58.13, 42.75, 38.60, 32.14, 27.89.

**Synthesis of HOP:**

A mixture of (E)-4-(2-(3-(dicyanomethylene)-5,5-dimethylcyclohex-1-en-1-yl)vinyl)-2-((((2-(5-hydroxy-1H-indol-3-yl)ethyl)carbamoyl)oxy)methyl)phenyl trifluoromethanesulfonate (1 g, 1.53 mmol), KOAc (450 mg, 4.59 mmol), Pd(dppf)Cl_2_ (100 mg, 0.153 mmol), and BINAP (583 mg, 2.295 mmol) in dry dioxane (20 mL) was degassed by alternating vacuum and argon purging three times. The reaction mixture was stirred at 90 °C for 5 h under an argon atmosphere. Upon completion, the reaction was cooled to room temperature and partitioned between ethyl acetate and water. The organic layer was washed with brine, dried over anhydrous MgSO₄, filtered, and concentrated under reduced pressure. The crude residue was purified by silica gel column chromatography using a 1:1 mixture of PE and EA as the eluent, yielding **HOP** as an orange solid (220 mg, 22.8% yield). ^1^H NMR (300 MHz, DMSO-*d_6_*) δ 10.46 (s, 1H), 8.58 (s, 1H), 7.72 (d, *J* = 7.4 Hz, 2H), 7.67 (s, 1H), 7.47 (d, *J* = 16.1 Hz, 1H), 7.36-7.22 (m, 2H), 7.12 (d, *J* = 8.6 Hz, 1H), 7.04 (s, 1H), 6.92 (s, 1H), 6.83 (s, 1H), 6.59 (d, *J* = 6.7 Hz, 1H), 5.23 (s, 2H), 3.26 (d, *J* = 6.9 Hz, 2H), 2.84-2.68 (m, 2H), 2.61 (s, 2H), 2.54 (s, 2H), 1.30 (s, 12H), 1.01 (s, 6H). ^13^C NMR (75 MHz, DMSO-*d_6_*) δ 170.70, 156.52, 155.79, 150.63, 143.87, 138.81, 137.19, 136.42, 131.26, 128.33, 123.89, 123.46, 114.19, 113.37, 112.09, 111.73, 111.08, 102.63, 84.16, 77.48, 65.47, 41.55, 32.11, 27.88, 25.06. HRMS (m/z) calcd. for C_37_H_41_BN_4_O_5_ [M+H]^+^ 632.3170, found 633.3234.

1. ***Supplementary Tables and Figures***

**
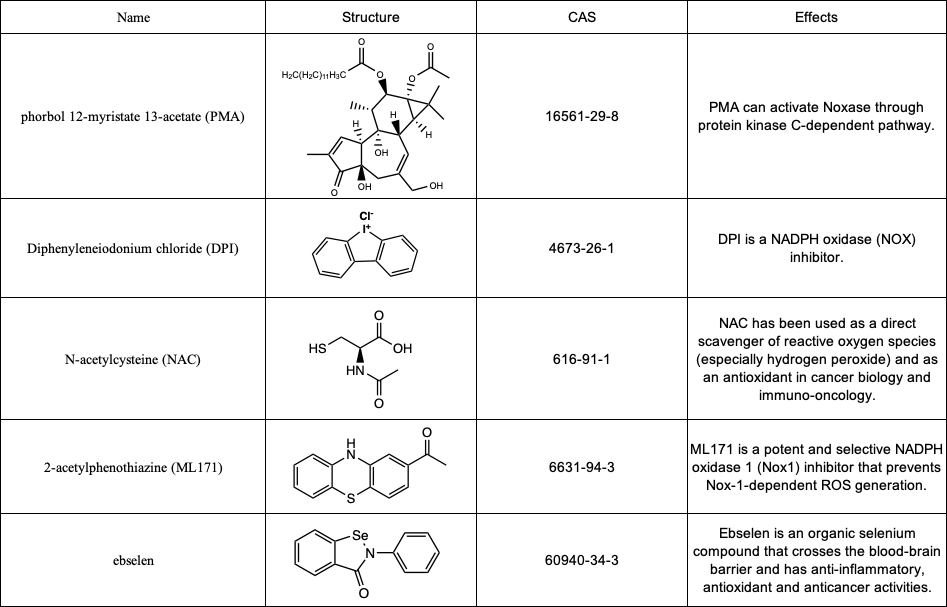
**

**Table S1.** Effective compounds used in endogenous H_2_O_2_ detection assays.

**
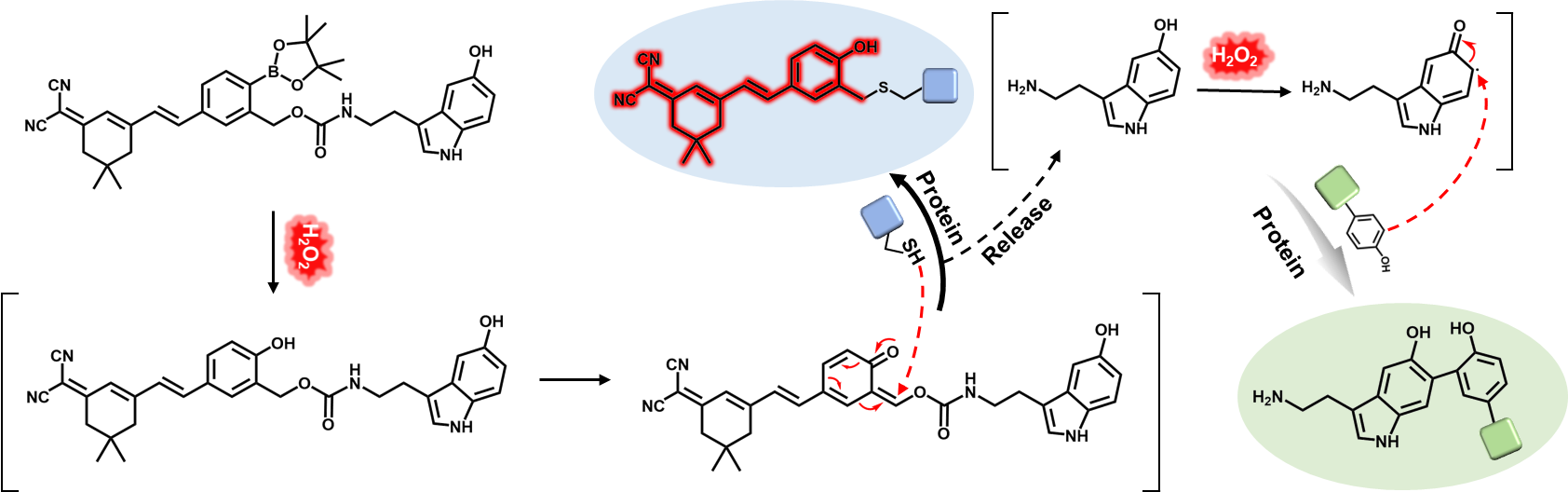
**

**Figure S1.** The way of covalent binding of **HOP** to nearby proteins after activation by H_2_O_2._


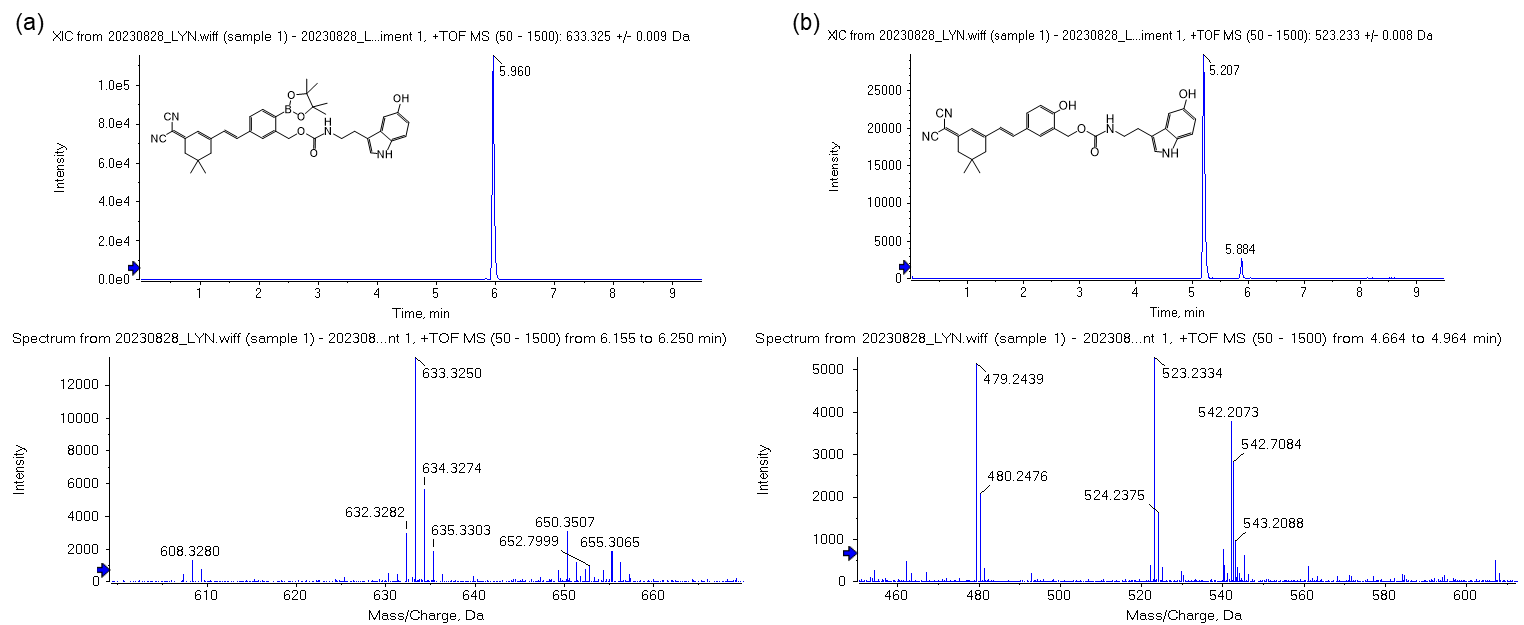


**Figure S2.** HPLC-MS studies of reaction mixtures. The LC-MS analysis of **HOP** before (a) and after (b) reaction with H_2_O_2_. LC-MS analysis of the mixture containing **HOP** (10 μM) and H_2_O_2_ (500 μM) was performed after incubation for 30 min at 37 °C in a buffer containing 50% water, 49% methanol and 1% DMSO.

\

**Figure S3**. Fluorescence response of **HOP** (5 μM) to different viscosities (H_2_O and Glycerol).


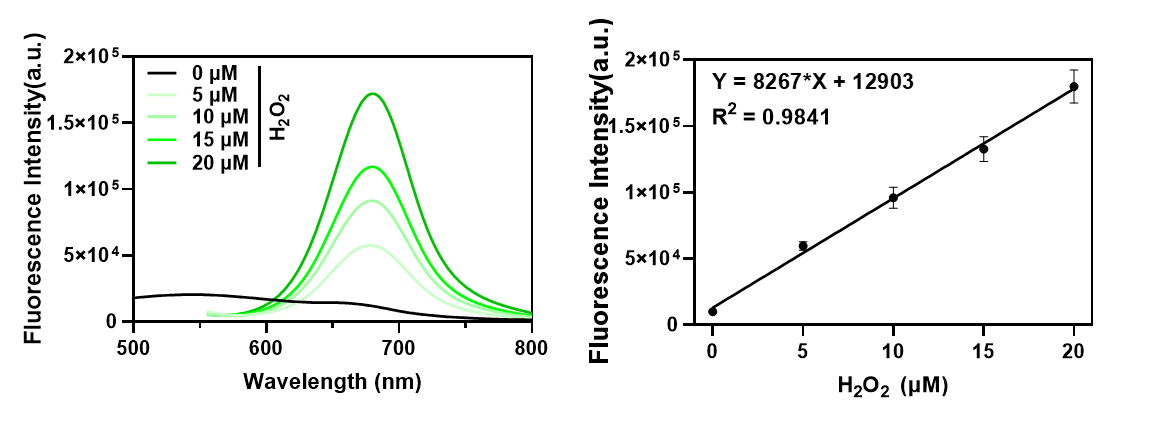


**Figure S4**. The linear correlation between fluorescence emission intensity of **HOP** and H_2_O_2_ concentration (0-20 μM). **HOP** (5 μM) was incubated with varying concentrations of H_2_O_2_ in PBS buffer (10 mM, pH 7.4, 1 mM CTAB) at 37 ℃ for 15 min. Fluorescence data were recorded with excitation at 535 nm, and the emission intensity was averaged over three independent experiments. The calculated limit of detection (LOD) was determined using the formula LOD = (3 × standard deviation)/slope (LOD =1118.851501*3/8267 =0.406 μM).

**Figure S5**. UV absorption spectra of **HOP** (10 μM) measured in H_2_O or octanol buffer (1% DMSO).


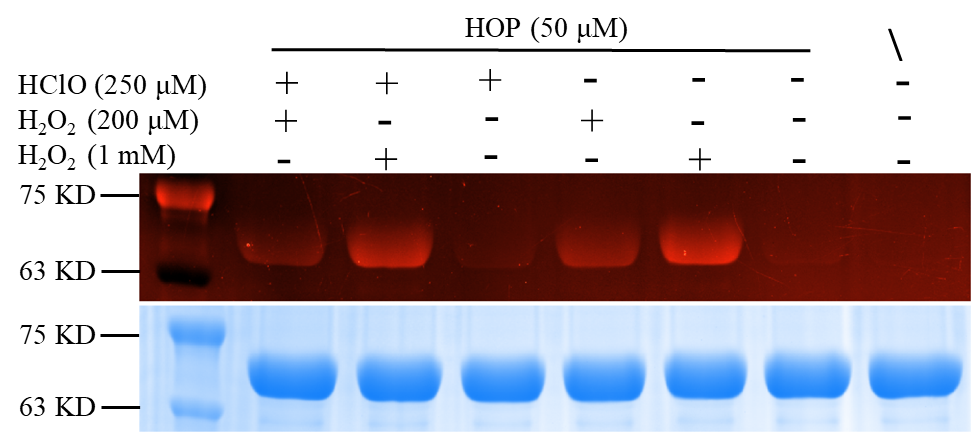


**Figure S6.** BSA (1 μg/μL, 10 μL) was incubated with **HOP** (50 μM) in the presence of different concentrations (200 μM and 1 mM) of H_2_O_2_ and HClO (250 μM) for 20 min, then separated by SDS-PAGE and analysed by in-gel fluorescence and Coomassie blue staining.


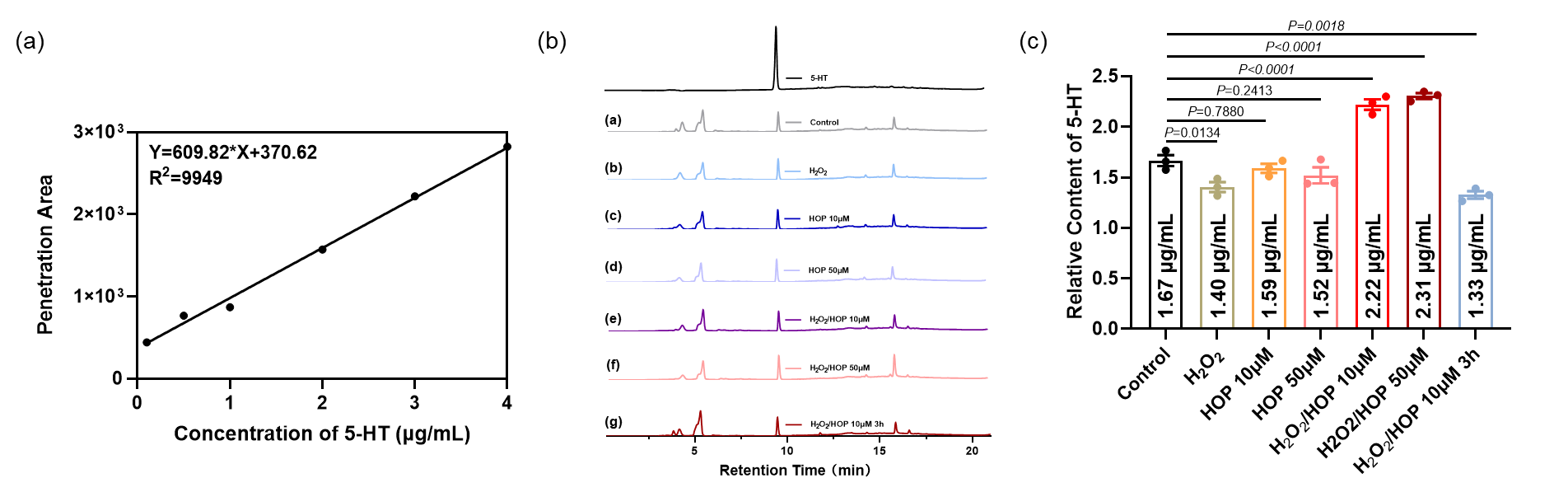


**Figure S7.** The HPLC quantitative analysis of 5-HT released from **HOP** in the microenvironment of cellular oxidative stress. (a) HPLC analysis of different concentrations of 5-HT standards by gradient dilution and the corresponding standard curves were plotted based on peak areas. (b) HPLC analysis of intracellular 5-HT content after cells were treated with different concentrations of **HOP** for different times in the presence or absence of H_2_O_2_, respectively. (c) Quantitative analysis of 5-HT content in (b). Statistical normalization was performed, with the 5-HT content of the untreated control group set to 1. Statistical significance was assessed using one-way ANOVA with multiple comparisons (^*^P < 0.05, ^**^P < 0.01, ^***^P < 0.001, ^****^P < 0.0001). Data are presented as mean ± SEM (n = 3).

**Figure S8.** Cytotoxicity assessment of **HOP** in GL261 cells. Cell viability of GL261 cells after 24 h treatment with different concentrations of **HOP**. Data are presented as mean ± SEM (n = 6).


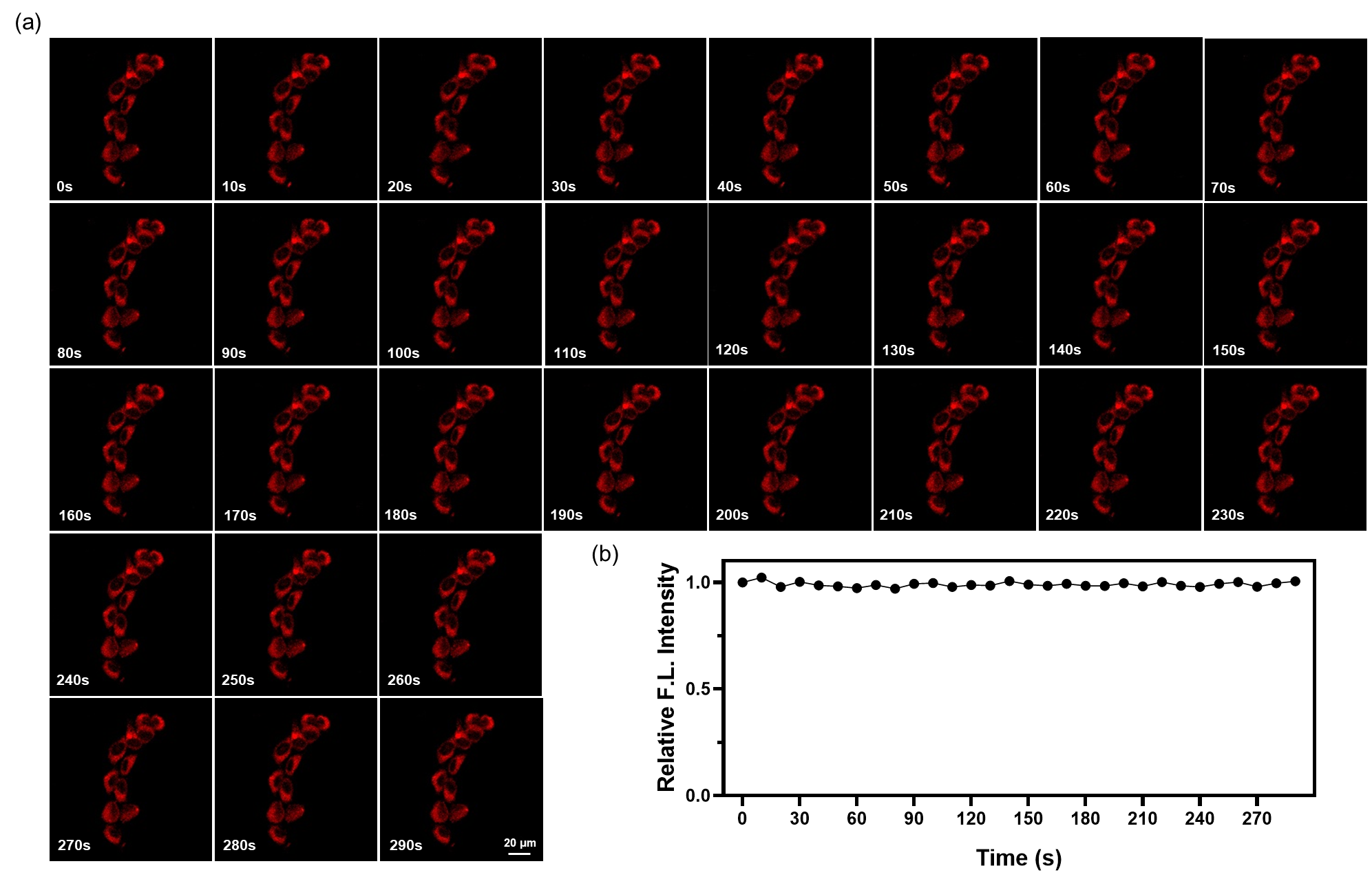


**Figure S9.** (a) Fluorescence images of cells incubated with **HOP** (5 μM) at different exposure times over 300s. (b) Changes in relative fluorescence intensity ratio with different exposure times. Where the cells were imaged every 10 s during 300 s, the excitation wavelength was 561 nm and the fluorescence images were acquired at 650-750 nm.


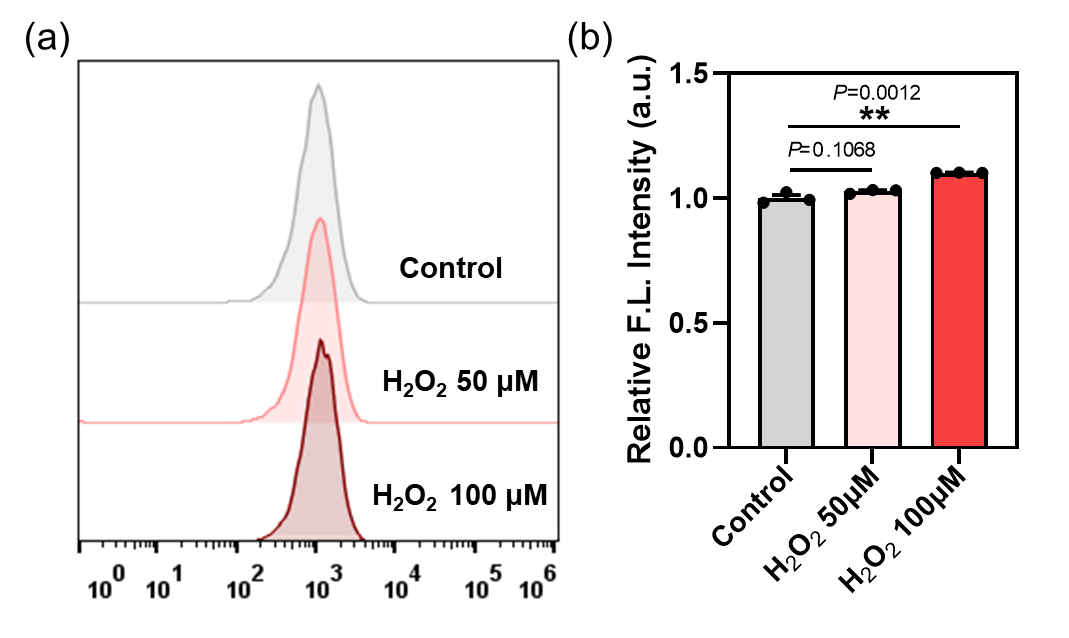


**Figure S10.** FACS analysis of **HOP** labeling in living GL261 cells. (a) Flow cytometry analysis of GL261 cells incubated with **HOP** (5 μM) for 20 min at 37 °C after treatment with varying concentrations of H₂O₂. Fluorescence was detected using the YL3 channel (excitation: 561 nm; emission: 655-735 nm). (b) Quantification of the relative fluorescence intensity ratios of the cells in (a), normalized to untreated cells (set as 1). Statistical analysis was performed using unpaired t-tests. Significance levels: ^*^*P* < 0.05, ^**^*P* < 0.01. Error bars represent mean ± SEM (n = 3).


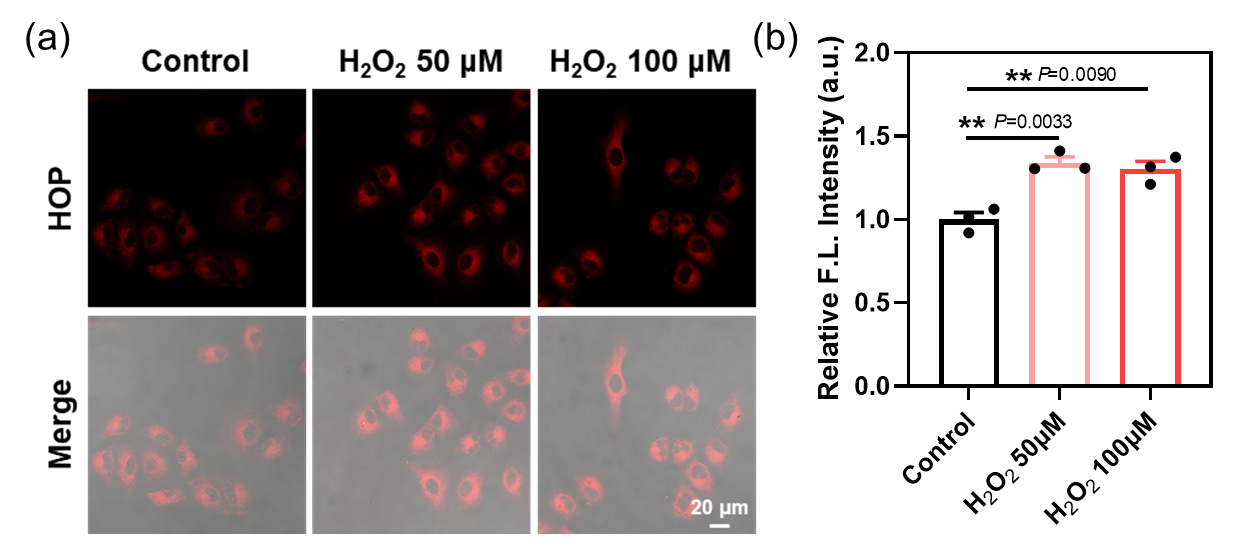


**Figure S11.** Confocal imaging analysis of **HOP** labeling in living GL261 cells. (a) Confocal microscopy images of GL261 cells incubated with **HOP** (5 μM) for 20 min at 37 °C following treatment with varying concentrations of H₂O₂. Fluorescence signals were collected from the emission channel (650-750 nm) with excitation at 561 nm. Scale bar = 20 μm. (b) Quantification of the relative fluorescence intensity ratios of the cells shown in (a), normalized to untreated cells (set as 1). Statistical analysis was performed using unpaired t-test. Significance levels: ^*^*P* < 0.05, ^**^*P* < 0.01. Error bars represent mean ± SEM (n = 3).


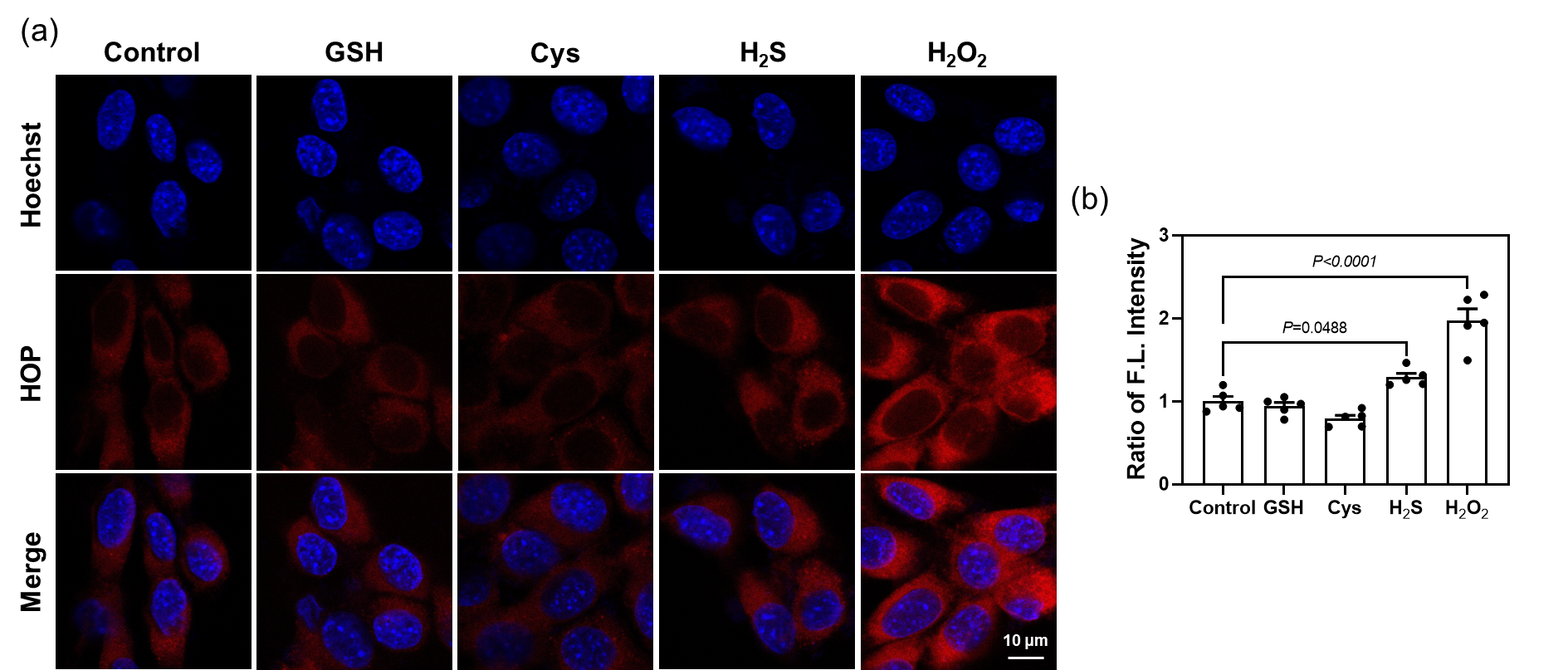


**Figure S12.** Confocal imaging analysis after treatment with different thiol small molecules. (a) Confocal microscopy images of HT22 cells treated with GSH, Cys, H_2_S and H_2_O_2_ (200 μM) for 30 min and then co-incubated with **HOP** (5 μM) for 20 min at 37℃. Fluorescence signals were collected from the emission channel (650-750 nm) with an excitation wavelength of 561 nm. scale=10 μm.(b) Quantification of the relative fluorescence intensity ratios of the cells shown in (a), normalised to that of untreated cells (set to 1). Statistical analyses were performed using one-way ANOVA and multiple comparisons. Significance levels: ^*^p < 0.05, ^**^p < 0.01, ^***^p < 0.001, ^****^p < 0.0001. Error bars represent mean ± SEM (n = 5).


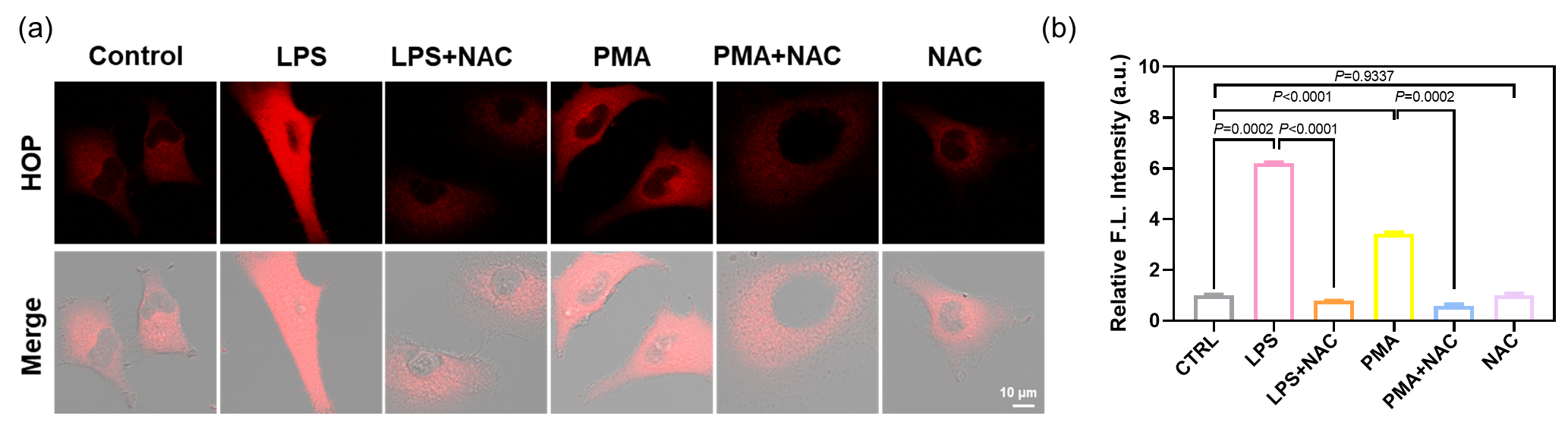


**Figure S13.** Confocal imaging analysis of **HOP** labeling in GL261 cells under different treatments. (a) Confocal microscopy images of GL261 cells incubated with **HOP** (5 μM) for 20 min at 37 °C after treatment with LPS (1 μg/mL, 12 h), PMA (1 μg/mL, 1 h), or NAC (2 mM, 30 min). Fluorescence signals were collected from the emission channel (650-750 nm) with excitation at 561 nm. Scale bar = 10 μm. (b) Quantification of the relative fluorescence intensity ratios of the cells shown in (a), normalized to untreated cells (set as 1). Statistical analysis was performed using one-way ANOVA with multiple comparisons. Significance levels: ^*^*P* < 0.05, ^**^*P* < 0.01, ^***^*P* < 0.001, ^****^*P* < 0.0001. Error bars represent mean ± SEM (n = 3).


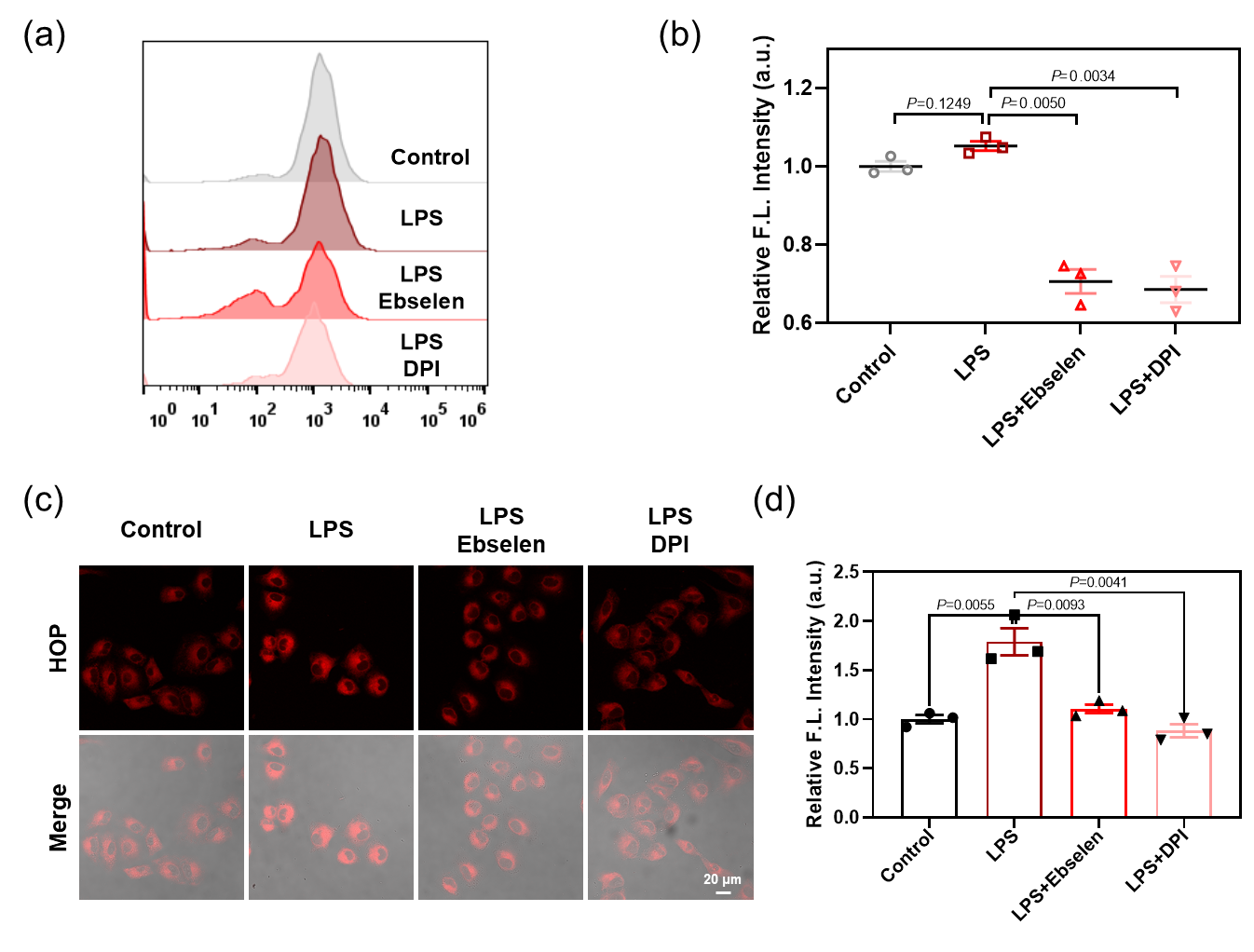


**Figure S14.** FACS and confocal imaging analysis of **HOP** labeling in SH-SY5Y cells under different treatments. (a) Flow cytometry analysis of SH-SY5Y cells incubated with **HOP** (5 μM) for 20 min at 37 °C after treatment with LPS (1 μg/mL, 12 h), Ebselen (5 μM, 30 min), or DPI (5 μM, 30 min). Fluorescence was detected in the emission channel (650-750 nm) with excitation at 561 nm. (b) Quantification of the relative fluorescence intensity ratios of the cells shown in (a), normalized to untreated cells (set as 1). (c) Confocal microscopy images of SH-SY5Y cells under the same conditions as in (a). Scale bar = 20 μm. (d) Quantification of the relative fluorescence intensity ratios of the cells shown in (c), normalized to untreated cells (set as 1). Statistical analysis was performed using unpaired t-tests. Significance levels: ^*^*P* < 0.05, ^**^*P* < 0.01. Error bars represent mean ± SEM (n = 3).


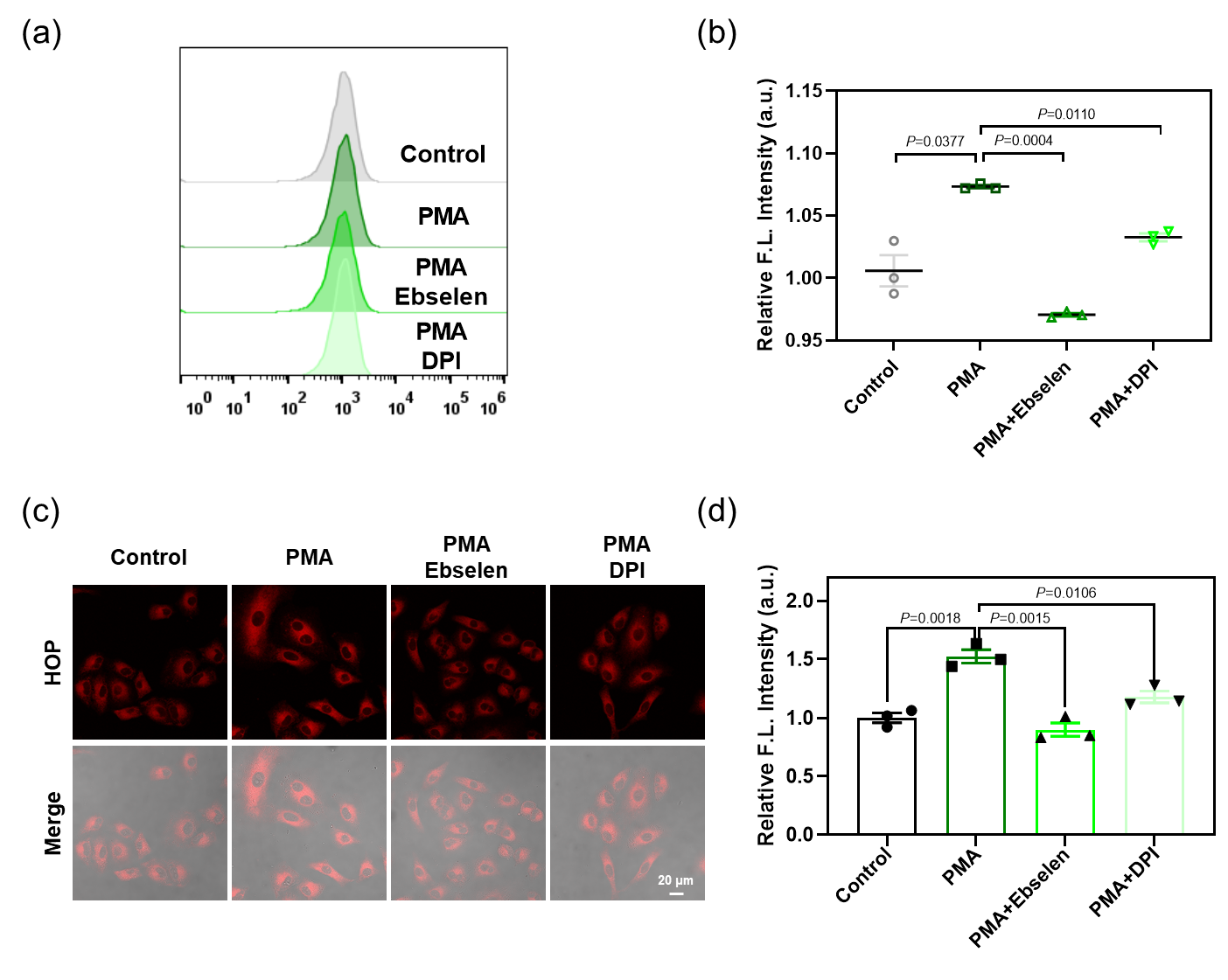


**Figure S15.** FACS and confocal imaging analysis of **HOP** labeling in SH-SY5Y cells under different treatments. (a) Flow cytometry analysis of SH-SY5Y cells incubated with **HOP** (5 μM) for 20 min at 37 °C following treatment with PMA (1 μg/mL, 1 h), Ebselen (5 μM, 30 min), or DPI (5 μM, 30 min). Fluorescence was detected in the emission channel (650-750 nm) with excitation at 561 nm. (b) Quantification of the relative fluorescence intensity ratios of the cells shown in (a), normalized to untreated cells (set as 1). (c) Confocal microscopy images of SH-SY5Y cells under the same conditions as in (a). Scale bar = 20 μm. (d) Quantification of the relative fluorescence intensity ratios of the cells shown in (c), normalized to untreated cells (set as 1). Statistical analysis was performed using paired t-tests. Significance levels: ^*^*P* < 0.05, ^**^*P* < 0.01, ^***^*P* < 0.001. Error bars represent mean ± SEM (n = 3).


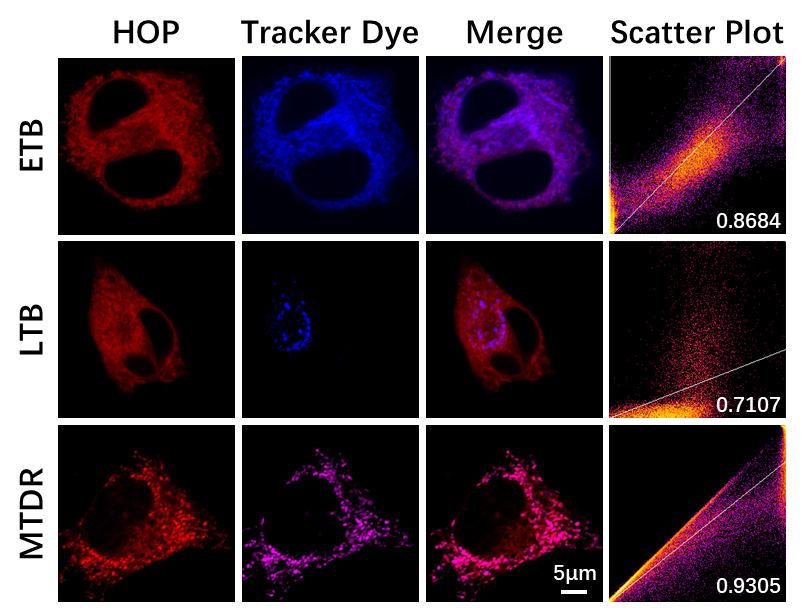


**Figure S16.** Airyscan imaging of intracellular colocalization in GL261 cells. Cells were incubated with **HOP** (5 μM, 20 min), followed by staining with Mito-Deep Red (100 nM, 30 min), ER-Blue (1 μM, 20 min), and Lyso-Blue Trackers (60 nM, 60 min). Fluorescence images were recorded under the following conditions. The **HOP** channels were acquired between 650-750 nm upon excitation at 561 nm. Tracker-Blue channels were acquired between 410-600 nm upon excitation at 405 nm. Tracker-Deep Red channel was acquired between 650-700 nm upon excitation at 633 nm. Scale = 5 μm. Pearson's correlation coefficient was calculated using Image J software.


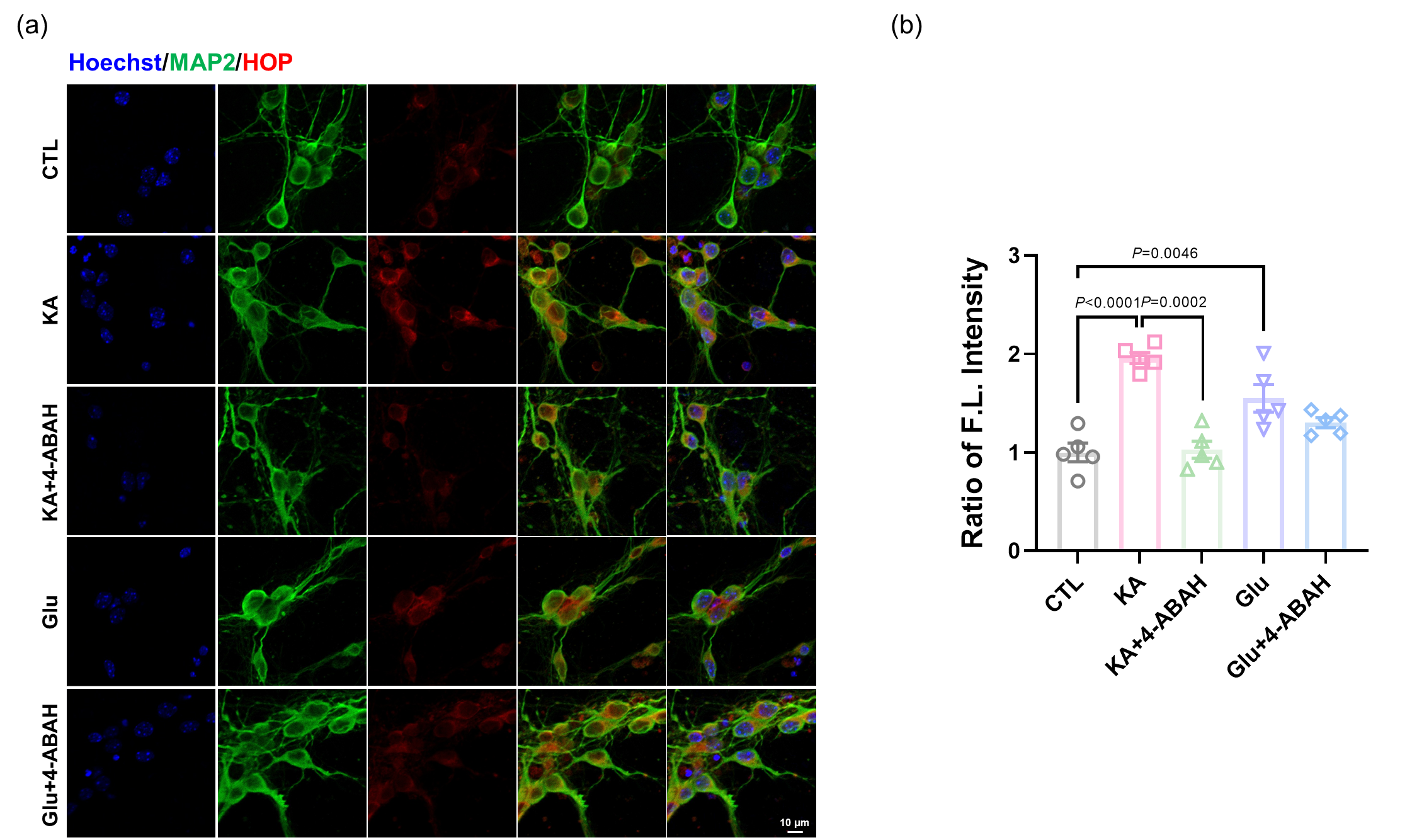


**Figure S17.** Confocal microscopy analysis of live-cell labeling in primary neurons using **HOP**. (a) Primary neurons were treated with KA (500 μM, 1 h), Glu (2 mM, 1 h), and 4-ABAH (500 μM, 1 h), followed by incubation with **HOP** (5 μM) at 37 °C for 20 min. Fluorescence signals of **HOP** were were collected from the emission channel at 650-750 nm, with excitation at 561 nm. For MAP2 labeling, the excitation wavelength was 488 nm, and emission was collected between 500 and 550 nm. Scale bar, 10 μm. (b) Quantification of the fluorescence intensity ratios of the labeled cells shown in panel (a). Untreated cells served as controls and were assigned a relative fluorescence intensity of 1. Data are presented as mean ± SEM (n = 5). Statistical significance was assessed using one-way ANOVA with multiple comparisons; ^*^*P* < 0.05, ^**^*P* < 0.01, ^***^*P* < 0.001, ^****^*P* < 0.0001.


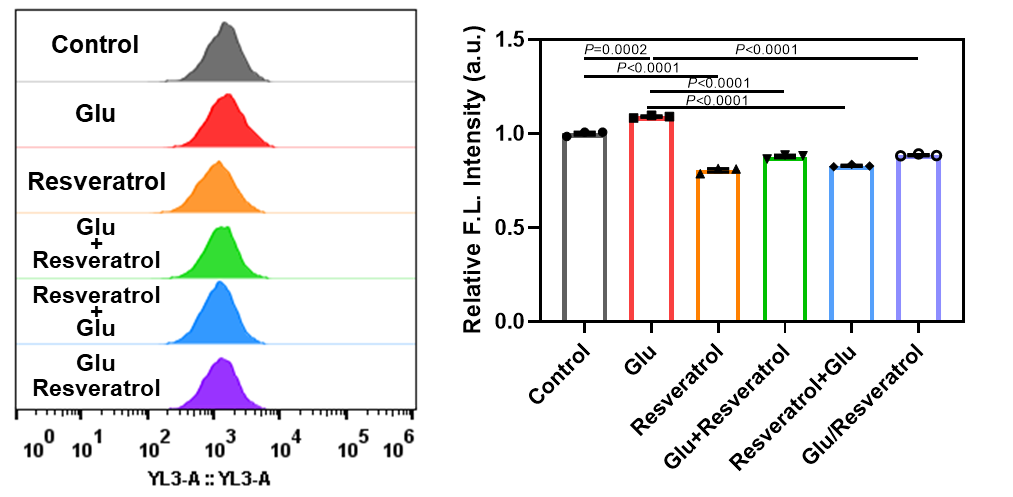


**Figure S18.** FACS analysis of live-cell labeling in primary neural cells using **HOP**. Primary neural cells were treated with Glu (2 mM, 1 h) or Resveratrol (1 μM, 1 h), followed by incubation with **HOP** (5 μM) for 20 min at 37 °C. Cells were then analyzed by flow cytometry using the YL3 channel (excitation: 561 nm, emission: 655-735 nm). The relative fluorescence intensities of the cells were quantified, with untreated cells serving as the control (set to 1). Statistical analyses were performed using one-way ANOVA with multiple comparisons. *P* < 0.05 was considered statistically significant (^*^*P* < 0.05, ^**^*P* < 0.01, ^***^*P* < 0.001, ^****^*P* < 0.0001). Data are presented as mean ± SEM (n = 3).

**Figure S19.** Quantification of SIRT1 protein levels in Figure 5D. SIRT1 protein expression in the PBS-treated control group was normalized to 1. Statistical significance was assessed using one-way ANOVA with multiple comparisons (^*^*P* < 0.05, ^**^*P* < 0.01, ^***^*P* < 0.001, ^****^*P* < 0.0001). Data are presented as mean ± SEM (n = 3).


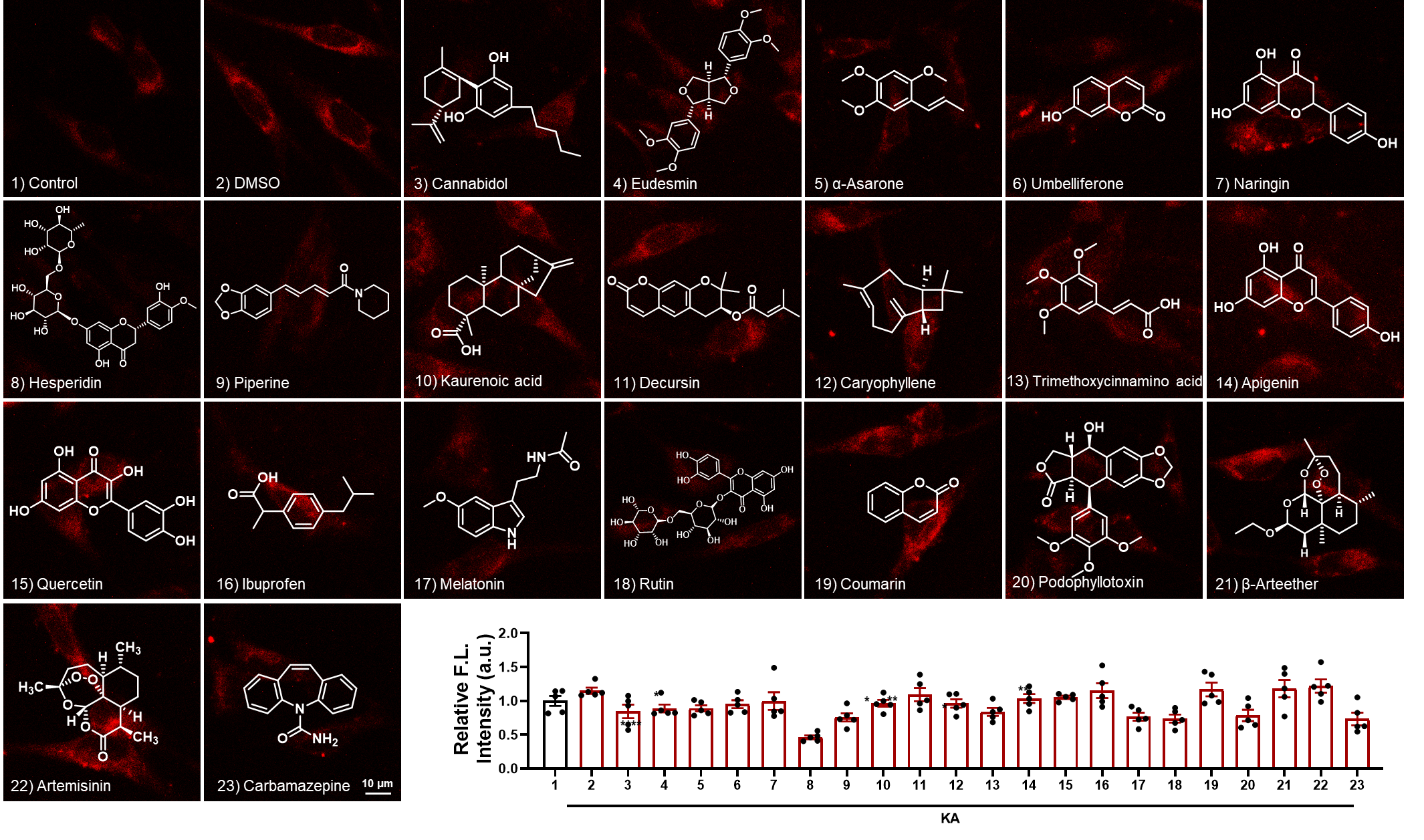


**Figure S20.** High-throughput screening of natural products with **HOP** in living HT22 mouse neuronal cells to control oxidative stress induced by KA. (A) HT22 cells were pretreated with KA (500 µM) for 1 h, followed by incubation with various natural antioxidants for 1 h. Afterward, cells were incubated with **HOP** (5 μM) for 20 min, and images were acquired using high-throughput analysis (scale bar, 10 μm). The relative fluorescence intensity was quantified. Blank represents cells without any treatment; Control represents cells treated with KA and without incubation of any natural products. The fluorescence intensity of the Blank group was set to 1. The excitation wavelength was 561 nm, and fluorescence emission was measured between 650 and 750 nm. Statistical significance was assessed using one-way ANOVA with multiple comparisons (^*^*P* < 0.05, ^**^*P* < 0.01, ^***^*P* < 0.001, ^****^*P* < 0.0001). Data are presented as mean ± SD (n = 5).


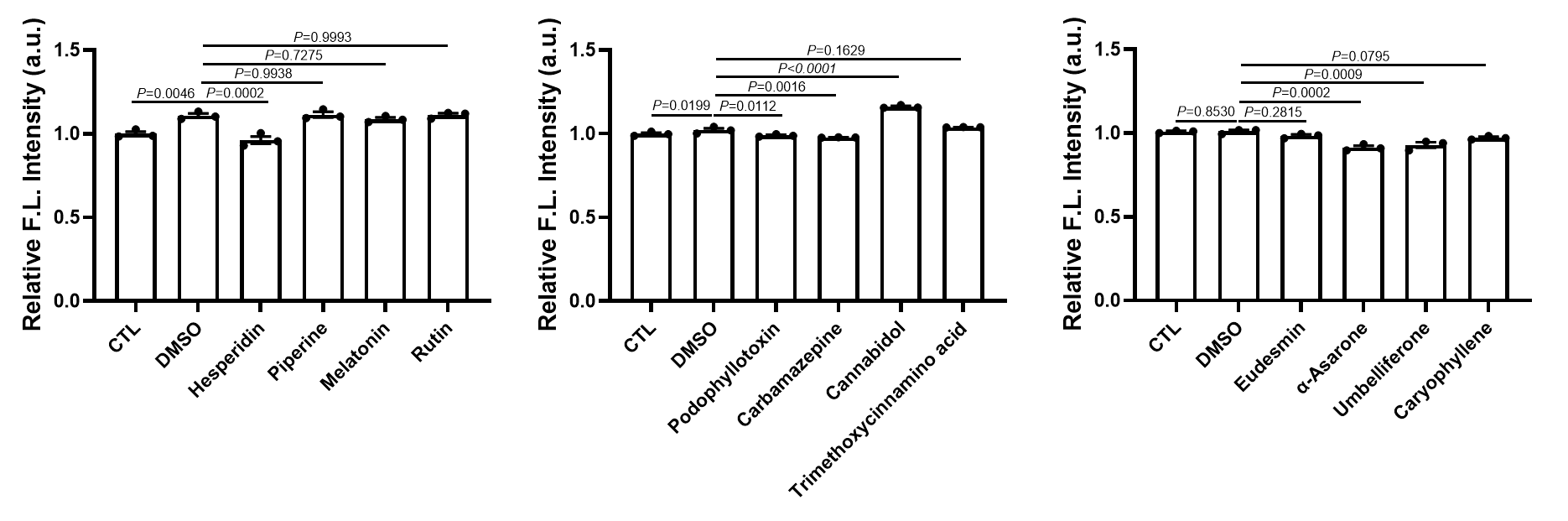


**Figure S21.** Flow cytometry analysis of 12 natural products initially screened using high-throughput screening. HT22 cells were pretreated with KA (500 µM) for 1 h, followed by incubation with various natural antioxidants for 1 h. Cells were then incubated with **HOP** (5 μM) for 20 min at 37 °C and analyzed by flow cytometry using the YL3 channel (excitation: 561 nm, emission: 655-735 nm). The relative fluorescence intensities were quantified, with untreated cells set to 1. Statistical analyses were performed with one-way ANOVA with multiple comparisons (^*^*P* < 0.05, ^**^*P* < 0.01, ^***^*P* < 0.001, ^****^*P* < 0.0001). Data are presented as mean ± SEM (n = 3).


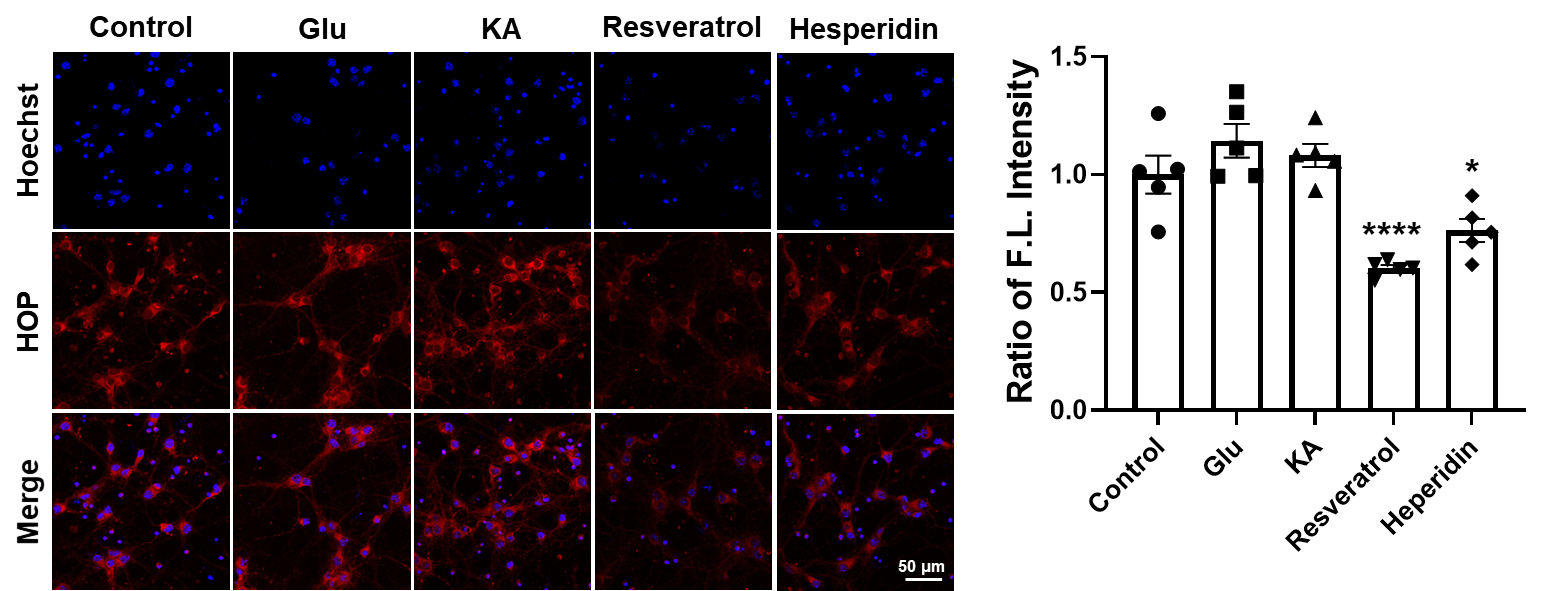


**Figure S22.** Confocal microscopy analysis of live-cell labeling in primary neural cells with **HOP**. Primary neural cells were treated with Glu (2 mM, 1 h), KA (500 μM, 1 h), Resveratrol (1 μM, 1 h), or Hesperidin (20 μM, 1 h), and then incubated with **HOP** (5 μM) for 20 min at 37 °C. Fluorescence signals were collected from the emission channel (650-750 nm) with excitation at 561 nm. Hoechst staining was used for nuclear labeling, with an excitation wavelength of 405 nm and emission collected between 420 and 480 nm. Scale bar, 50 μm. The relative fluorescence intensity ratios of the cells were quantified, with untreated cells set to 1. Statistical analyses were performed using one-way ANOVA with multiple comparisons. *P*< 0.05 was considered statistically significant (^*^*P* < 0.05, ^**^*P* < 0.01, ^***^*P* < 0.001, ^****^*P* < 0.0001). Data are presented as mean ± SEM (n = 5).


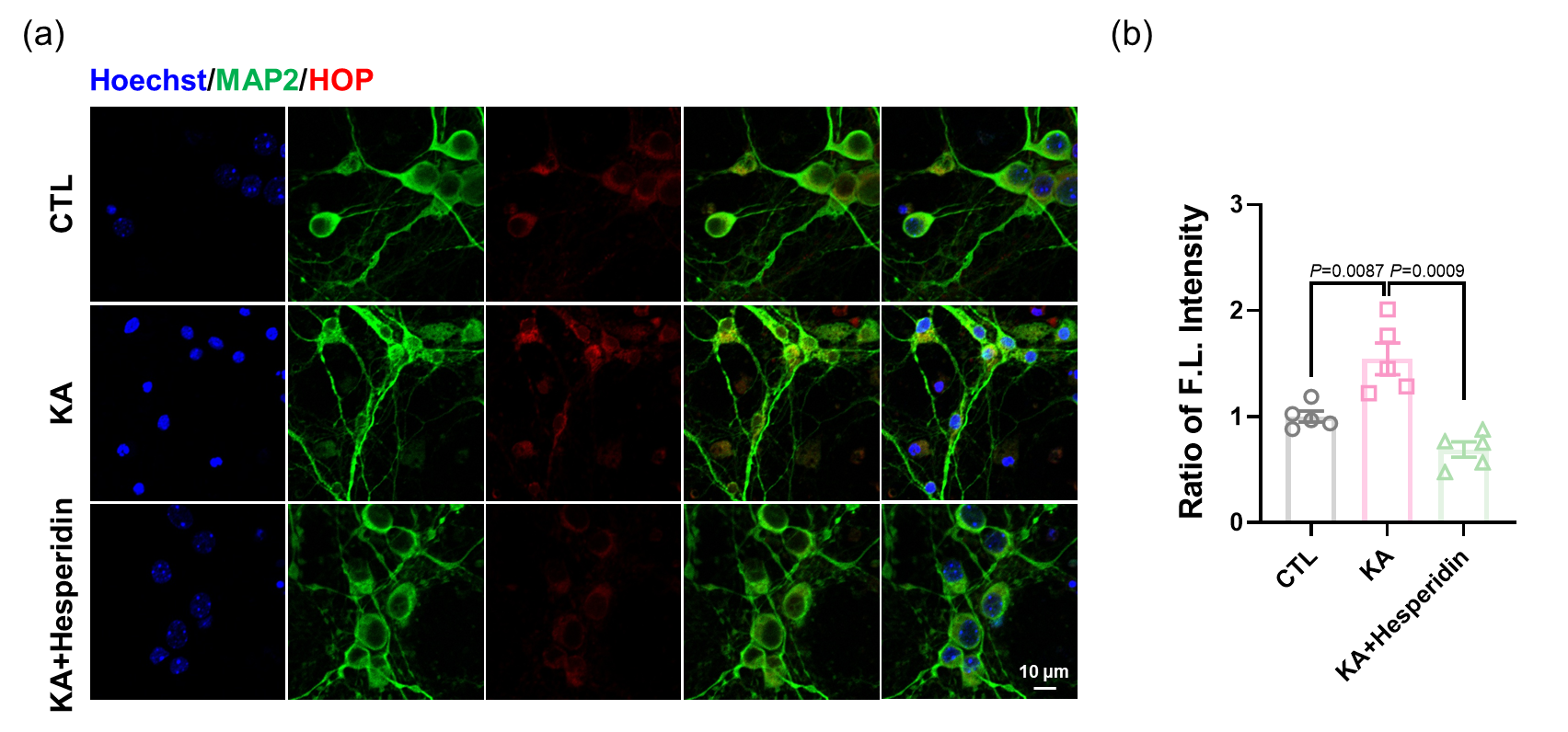


**Figure S23.** Confocal imaging analysis of living cell labeling of primary neural cells with **HOP**. (a) Living primary neural cells treated with KA (500 μM, 1 h) and Hesperidin (20 μM, 1 h), respectively, were incubated with **HOP** (5 μM) at 37 °C for 20 min, and then the fluorescence signals were collected from the emission channel at 650-750 nm with excitation at 561 nm. The excitation wavelength of Hoechst was 405 nm and the emission collection range was 420-480 nm. The excitation wavelength of MAP2 was 488 nm with the emission collected between 500 and 550 nm. Scale bar, 10 μm. (b) Quantification of the relative ratios of fluorescence intensities of the cells in (a). Cells without any treatment were set to 1. Statistical analyses were performed with one-way ANOVA with multiple comparisons. *P* < 0.05 is considered significant (^*^*P* < 0.05, ^**^*P* < 0.01, ^***^*P* < 0.001, ^****^*P* < 0.0001). Error bars are ±SEM (n = 5).


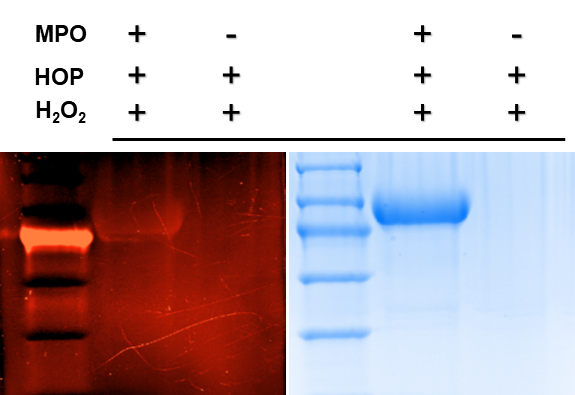


**Figure S24.** In-gel fluorescence analysis of MPO treatment with **HOP** in the presence of H_2_O_2_. MPO (1 μg/μL, 5 μL) was incubated with 20 μM **HOP** in the presence of 200 μM H_2_O_2_ for 20 min, then separated by SDS-PAGE and analyzed using in-gel fluorescence and Coomassie blue staining.


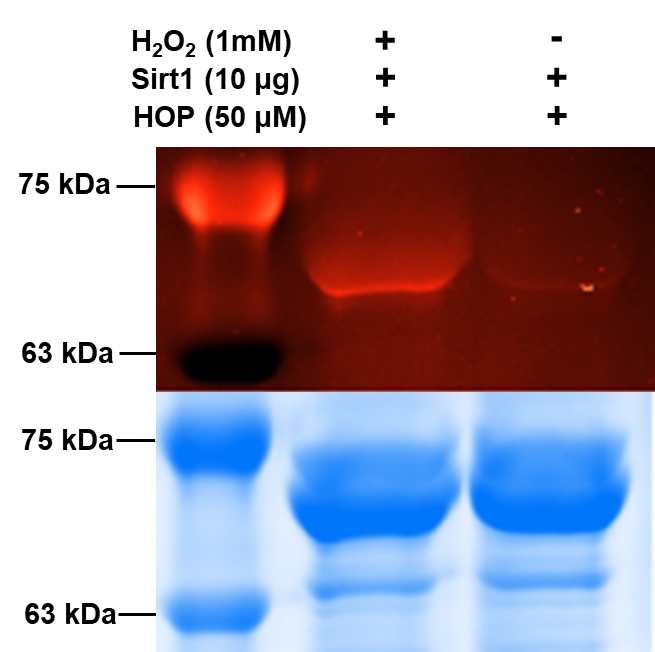


**Figure S25.** In-gel fluorescence analysis of SIRT1 treatment with **HOP** in the presence of H_2_O_2_. SIRT1 (1 μg/μL, 10 μL) was treated with 50 μM **HOP** in the presence of 1 mM H_2_O_2_ for 20 min, then separated by SDS-PAGE and analyzed by In-gel fluorescence and Coomassie blue staining.


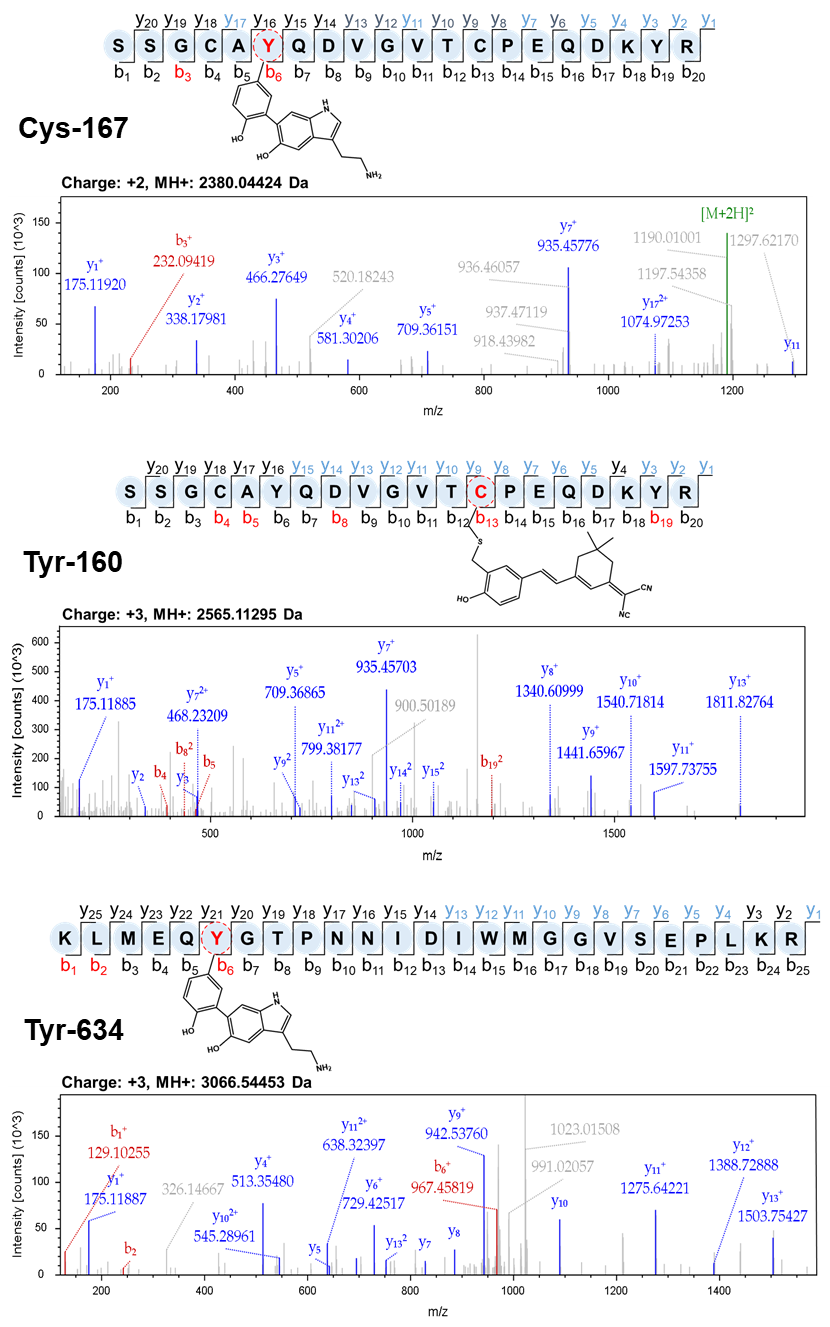


**Figure S26.** Identification of peptides modified by **HOP** using mass spectrometry. Mass spectrometry analysis identified two major peptides modified by **HOP**: SSGCAYQDVGVTCPEQDKYR and KLMEQYGTPNNIDIWMGGVSEPLKR. These results suggest that Cys-167, Tyr-160, and Tyr-634 are the primary binding sites for MPO.


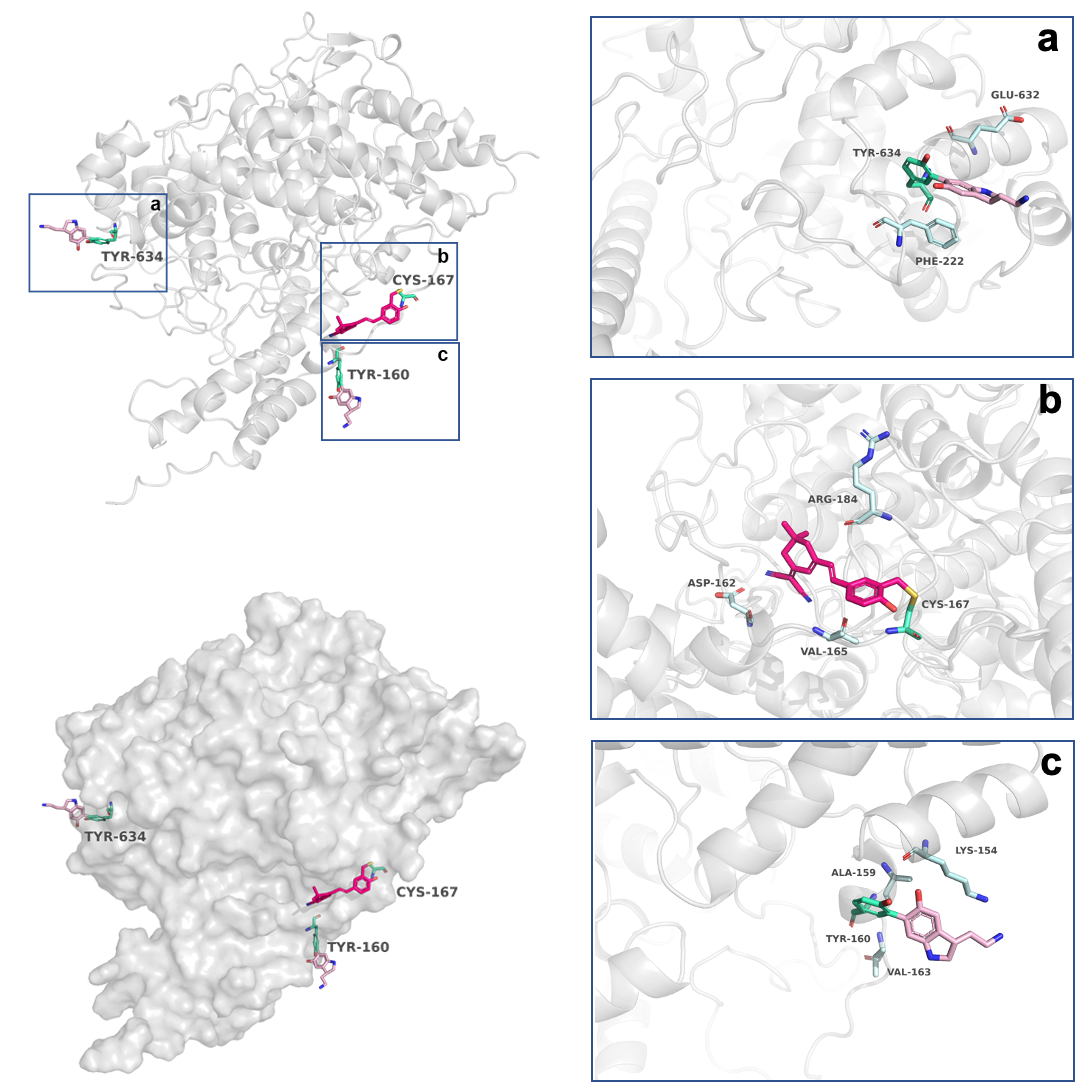


**Figure S27.** Analysis of the binding sites between MPO and **HOP**. The docking analysis of **HOP** covalently bound to MPO reveals three distinct binding sites. Panels (a), (b), and (c) show the simulated binding interactions of **HOP** with Tyr634, Cys167, and Tyr160, respectively.


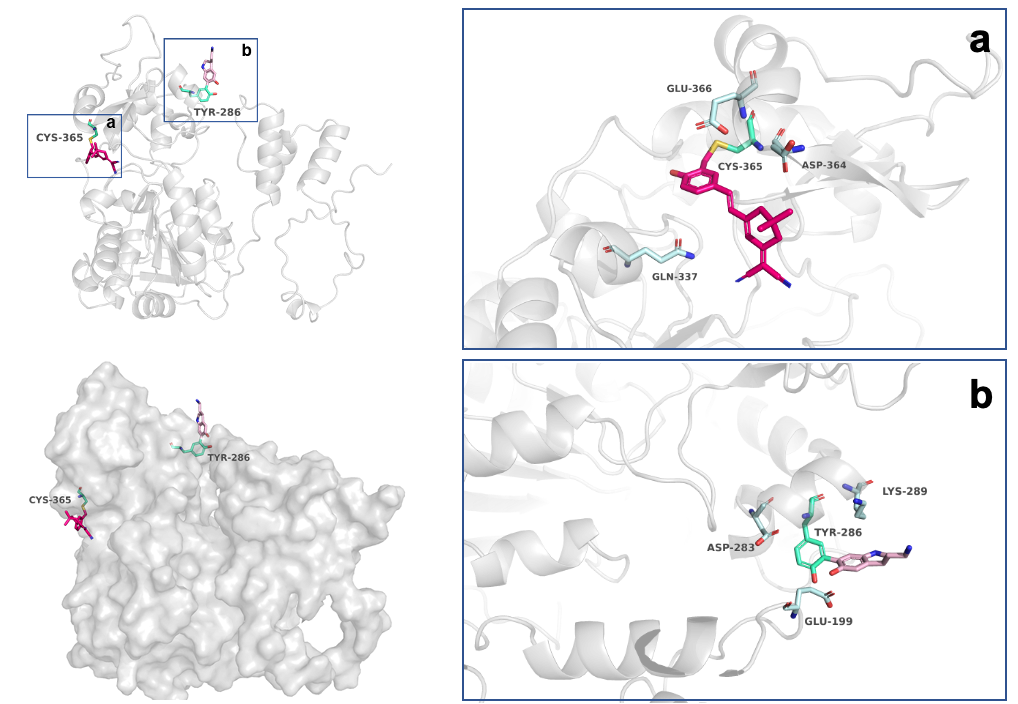


**Figure S28.** Analysis of the binding sites between SIRT1 and **HOP**. Docking analysis of **HOP** covalently bound to SIRT1 reveals two distinct binding sites. Panels (a) and (b) show the simulated binding interactions of **HOP** with Cys365 and Tyr286, respectively.


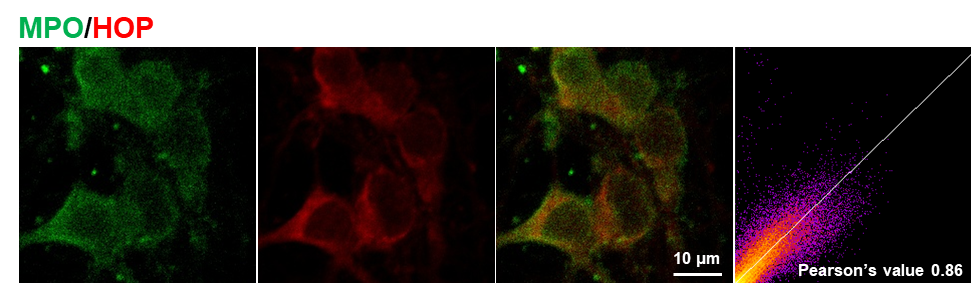


**Figure S29.** Immunofluorescence co-localization of **HOP** with MPO antibody. Primary neuronal cells were imaged after co-incubation with **HOP** (5 μM, 20 min), MPO antibody (Proteintech, 22225-1-AP, 1:500, 90 min), and Alexa Fluor 488 (Abclonal, AS073, 1:500, 60 min). Fluorescence signals for **HOP** were collected between 650-720 nm with excitation at 561 nm, and Tracker-Green signals were collected between 500-550 nm with excitation at 488 nm. Scale bar, 10 μm. Pearson's correlation coefficient was calculated using ImageJ.


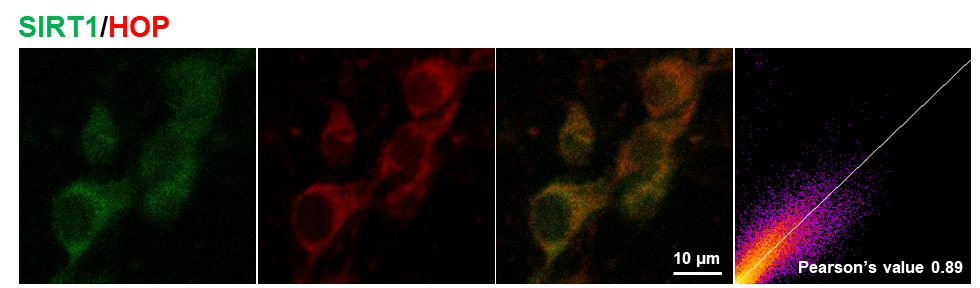


**Figure S30.** Immunofluorescence co-localization of **HOP** with SIRT1 antibody. Primary neuronal cells were imaged after co-incubation with **HOP** (5 μM, 20 min), SIRT1 antibody (Proteintech, 60303-1-Ig, 1:100, 90 min), and Alexa Fluor 488 (Beyotime, A0428, 1:500, 60 min). Fluorescence signals for **HOP** were collected between 650-720 nm with excitation at 561 nm, and Tracker-Green signals were collected between 500-550 nm with excitation at 488 nm. Scale bar, 10 μm. Pearson's correlation coefficient was calculated using ImageJ.


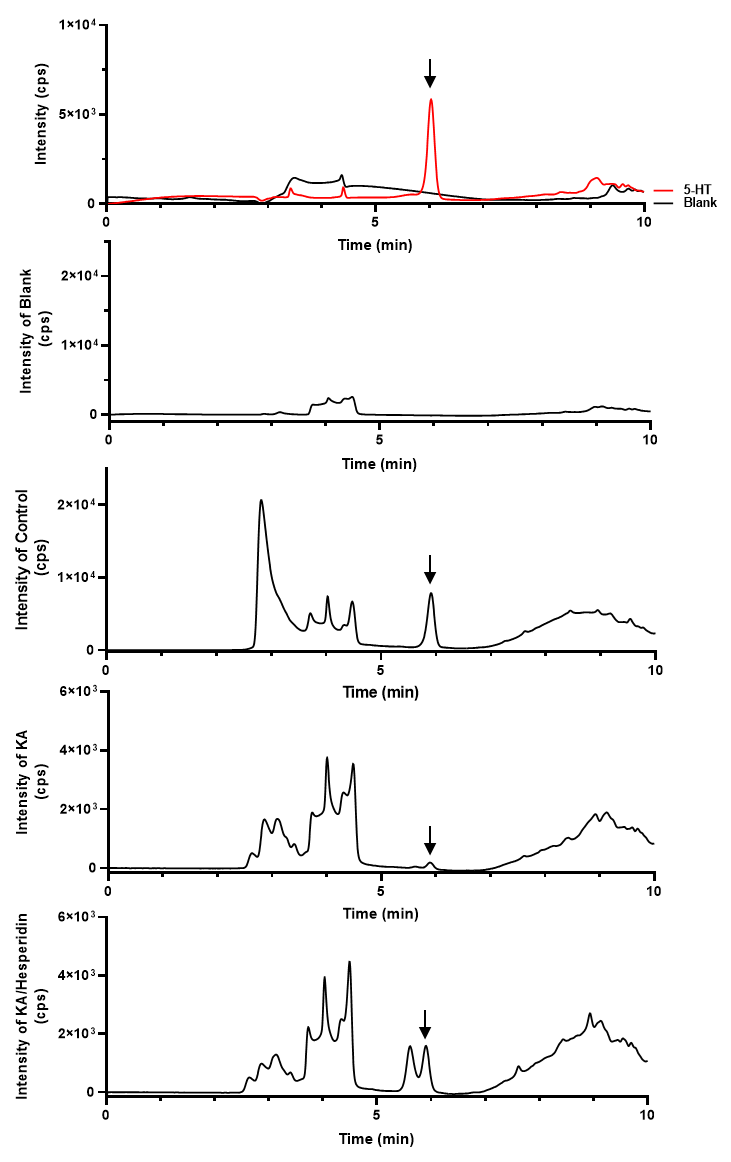


**Figure S31.** HPLC analysis of 5-HT levels in primary neuronal cells before and after hesperidin treatment in a KA-induced oxidative stress model. The HPLC profiles of 5-HT are shown for various conditions: (from top to bottom) 5-HT standard (2 μg/mL), blank samples, control samples, KA-induced samples, and samples from the group treated with hesperidin following KA-induced treatment.

**
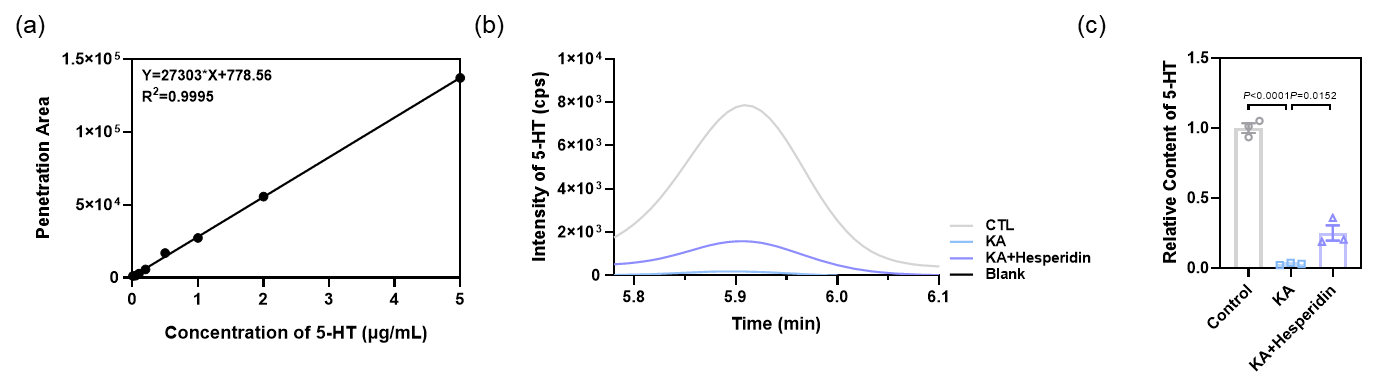
**

**Figure S32.** Determination of 5-HT content in primary neuronal cells across different treatment groups. (a) HPLC analysis of 5-HT standards at various concentrations, with a gradient dilution, and the corresponding standard curve developed from peak areas. (b) A magnified view of the 5-HT peak location in the HPLC profile that shown in **Figure S31**. (c) Peak areas from different cell samples in panel (b) were applied to the standard curve to calculate 5-HT content. Statistical normalization was performed, with the 5-HT content of the untreated control group set to 1. Statistical significance was assessed using one-way ANOVA with multiple comparisons (^*^*P* < 0.05, ^**^*P* < 0.01, ^***^*P* < 0.001, ^****^*P* < 0.0001). Data are presented as mean ± SEM (n = 3).


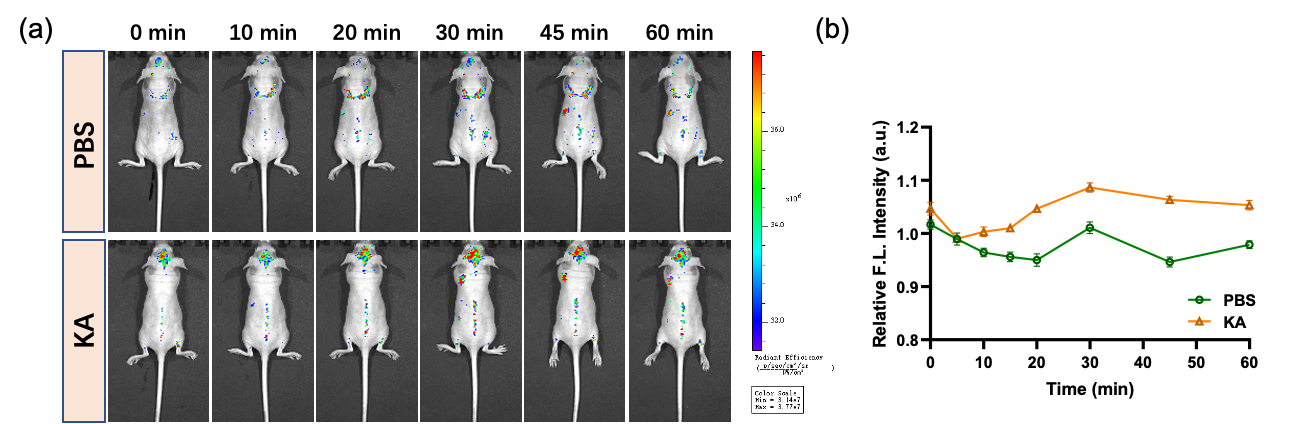


**Figure S33.** In vivo fluorescence imaging of the brain in KA-induced epileptic mice. (a) Fluorescence images of mice pre-treated with KA (6 mg/kg) for 12 h, followed by intravenous injection of **HOP**. Fluorescence was captured using an excitation wavelength of 560 nm and emission at 680 nm, measured post-injection at 10, 20, 30, 45, and 60 min. (b) Line plots showing the fluorescence intensity in the brains of control and epilepsy model mice over time.


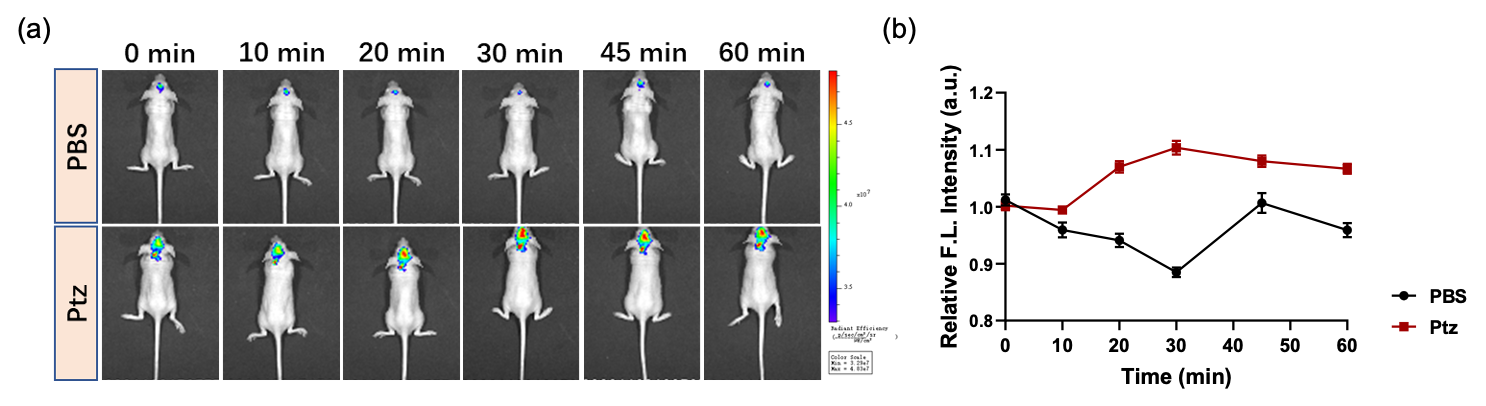


**Figure S34.** In vivo fluorescence imaging of the brain in Ptz-induced epileptic mice. (a) Fluorescence images of mice pre-treated with Ptz (60 mg/kg) for 12 h, followed by intravenous injection of **HOP**. Fluorescence was captured at an excitation wavelength of 560 nm and emission at 680 nm post-injection at 10, 20, 30, 45, and 60 min. (b) Line plots exhibit the fluorescence intensity in the brains of control and epilepsy model mice over time.

**Figure S35.** Time-course analysis of brain fluorescence intensity in control, Ptz-induced epileptic, and hesperidin-treated epileptic mice from Figure 6D.


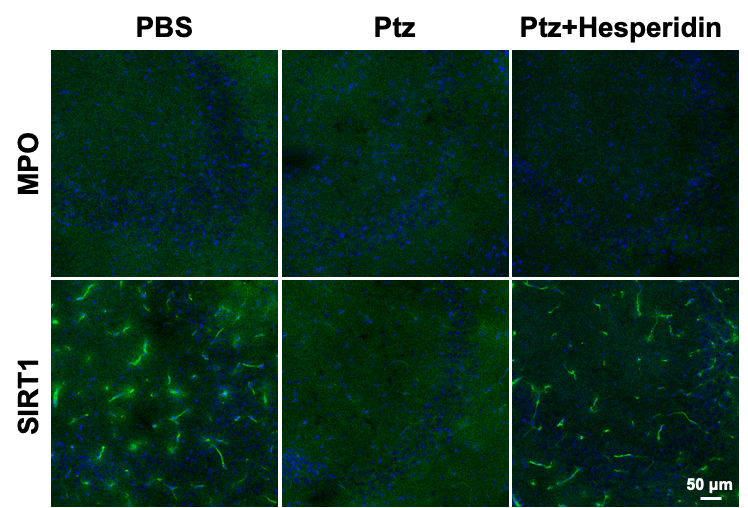


**Figure S36.** Immunofluorescence staining of MPO and SIRT1 proteins on frozen brain sections from normal, Ptz-induced epileptic mice, and hesperidin-treated epileptic mice. DAPI was excited at 405 nm with emission collected between 420-480 nm. Alexa Fluor 488-conjugated AffiniPure goat anti-rabbit/mouse IgG (H+L) was excited at 488 nm with emission collected between 500-550 nm. Scale bar, 50 μm.

**Figure S37.** Quantification of protein expression in the PBS group was normalized to 1. Statistical analyses were performed with one-way ANOVA with multiple comparisons. ^*^*P* < 0.05, ^**^*P* < 0.01, ^***^*P* < 0.001, ^****^*P* < 0.0001. Error bars are ±SEM (n = 3).


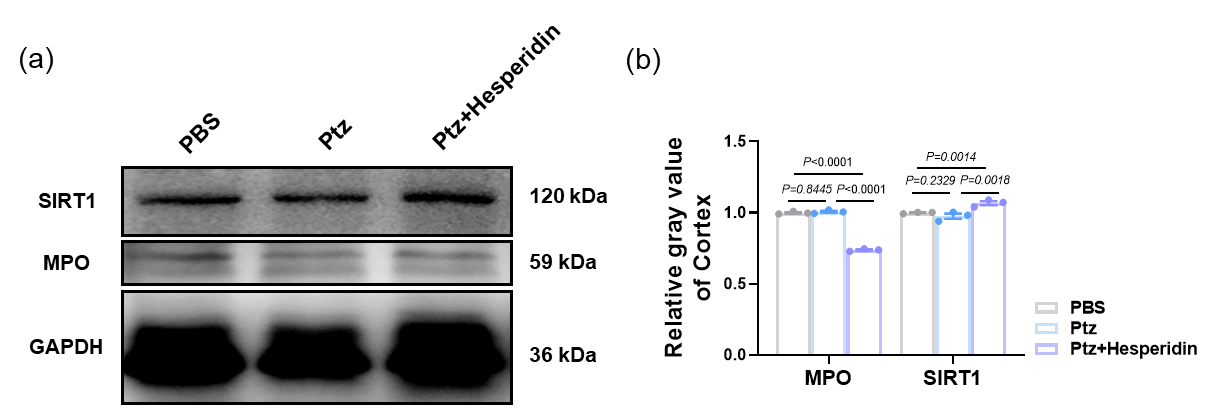


**Figure S38.** (a) The expression levels of MPO and SIRT1 of treated and control mice in cotex tissues were detected by Western blotting. (b) Quantification of protein expression levels in cotex tissues of shown in (a), with protein expression in the PBS group normalized to 1. Statistical significance was determined using one-way ANOVA with multiple comparisons (^*^*P* < 0.05, ^**^*P* < 0.01, ^***^*P* < 0.001, ^****^*P* < 0.0001). Data are presented as mean ± SEM (n = 3).

1. ***NMR Spectra & Mass Spectra***

**Figure S37.** ^1^H NMR spectra of compound **2** (CDCl_3_).

**Figure S35.** ^13^C NMR spectra of compound **2** (CDCl_3_).

**Figure S36.** ^1^H NMR spectra of compound **3** (CDCl_3_).

**Figure S37.** ^13^C NMR spectra of compound **3** (CDCl_3_).

**Figure S38.** ^1^H NMR spectra of compound **4** (DMSO-*d*_6_).

**Figure S39.** ^13^C NMR spectra of compound **4** (DMSO-*d*_6_).

**Figure S40.** ^1^H NMR spectra of compound **5** (DMSO-*d*_6_).

**Figure S41.** ^13^C NMR spectra of compound **5** (DMSO-*d*_6_).

**Figure S42.** ^1^H NMR spectra of compound **6** (DMSO-*d*_6_).

**Figure S43.** ^13^C NMR spectra of compound **6** (DMSO-*d_6_*).

**Figure S44.** ^1^H NMR spectra of compound **HOP** (DMSO-*d*_6_).

**Figure S45.** ^13^C NMR spectra of compound **HOP** (DMSO-*d*_6_).


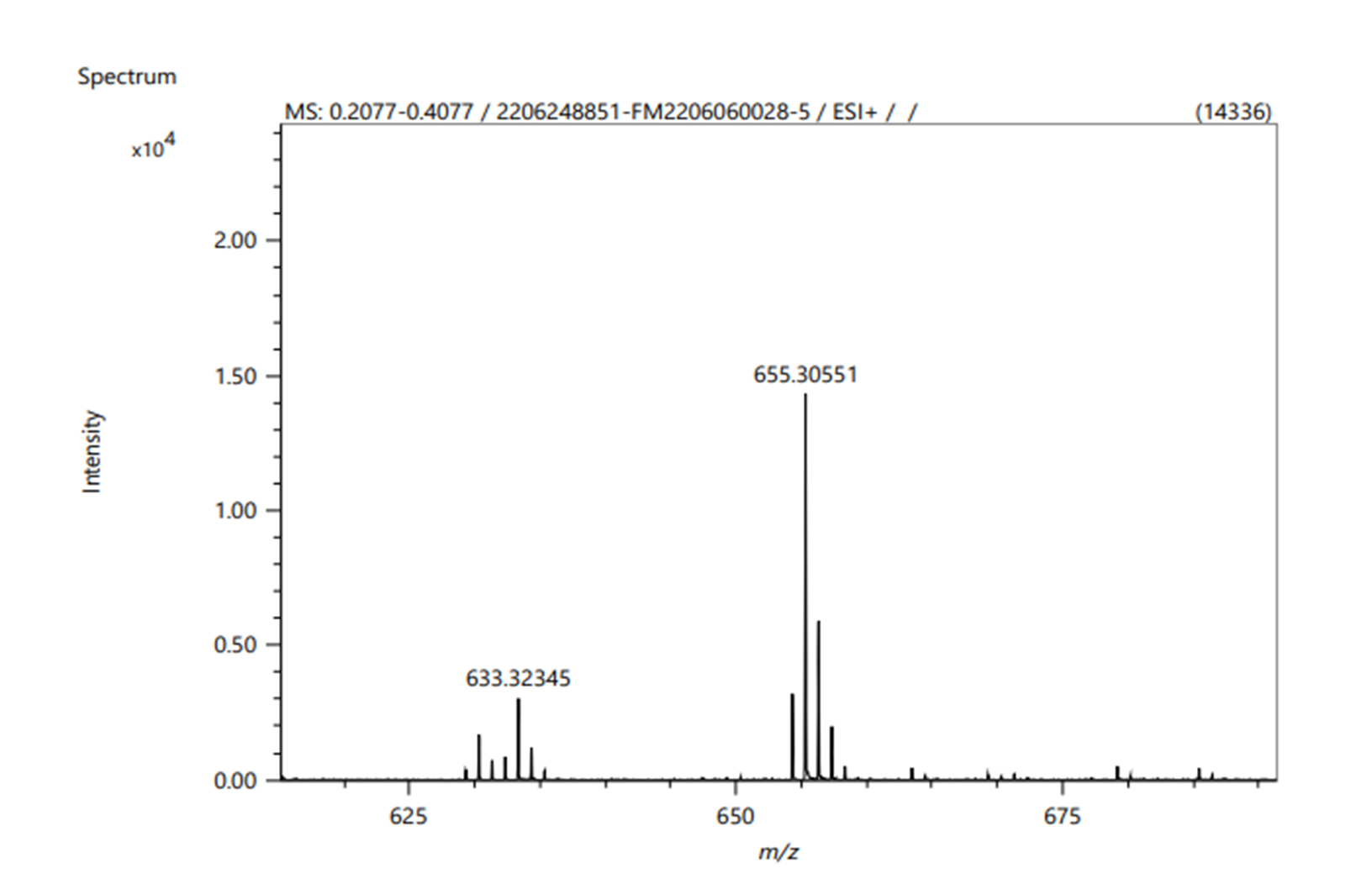


**Figure S46.** HR-MS spectra of **HOP**.
